# Prophage-encoded chitinase gene supports growth of its bacterial host isolated from deep-sea sediments

**DOI:** 10.1093/ismejo/wraf004

**Published:** 2025-01-20

**Authors:** Mathias Middelboe, Sachia J Traving, Daniel Castillo, Panos G Kalatzis, Ronnie N Glud

**Affiliations:** Marine Biological Section, Department of Biology, University of Copenhagen, Strandpromenaden 5, 3000 Helsingør, Denmark; HADAL & Nordcee, Department of Biology, University of Southern Denmark, Campusvej 55, 5230 Odense, Denmark; Marine Biological Section, Department of Biology, University of Copenhagen, Strandpromenaden 5, 3000 Helsingør, Denmark; HADAL & Nordcee, Department of Biology, University of Southern Denmark, Campusvej 55, 5230 Odense, Denmark; Marine Biological Section, Department of Biology, University of Copenhagen, Strandpromenaden 5, 3000 Helsingør, Denmark; Center for Evolutionary Hologenomics, Globe Institute, University of Copenhagen, Øster Farimagsgade 5, 1353 Copenhagen, Denmark; HADAL & Nordcee, Department of Biology, University of Southern Denmark, Campusvej 55, 5230 Odense, Denmark; Danish Institute for Advanced Study (DIAS), University of Southern Denmark, Campusvej 55, 5230 Odense, Denmark; Department of Ocean and Environmental Sciences, Tokyo University of Marine Science and Technology, 4-5-7 Konan, Minato-ku, 108-8477 Tokyo, Japan

**Keywords:** deep-sea sediment, auxiliary metabolic genes, cooperative goods, hydrostatic pressure, lysogeny, pseudomonas, biogeochemical cycling, Kermadec Trench, piezotolerant bacteria, chitinase

## Abstract

Auxiliary metabolic genes encoded by bacteriophages can influence host metabolic function during infection. In temperate phages, auxiliary metabolic genes (AMGs) may increase host fitness when integrated as prophages into the host genome. However, little is known about the contribution of prophage-encoded AMGs to host metabolic properties. In this study, we examined a temperate bacteriophage, and its piezotolerant *Pseudomonas* sp. host obtained from sediment samples collected from the Kermadec Trench at ~10 000 m water depth. Both the phage and host were present throughout the sediment profiles from the surface to 30 cm into the sediment, covering large gradients of environmental conditions. The host and phage each carried one chitinase gene, which differed from each other, suggesting that chitin degradation plays a role in their substrate supply. We demonstrated that prophage-encoded chitinase supported host chitin degradation and growth in the presence of chitin. Furthermore, prophage induction dynamics were strongly substrate-dependent, suggesting that the host controls the lysis-lysogeny switch in response to the presence of chitin, thus optimizing the trade-off between the loss of cells from prophage induction and prophage enhancement of host performance. Overall, the results demonstrate prophage-encoded AMGs as collaborative goods for their hosts and emphasize the potential role of phage-host interactions in benthic biogeochemical cycling, as well as for the capability of deep-sea bacteria to efficiently adapt and thrive at a wide range of environmental conditions.

## Introduction

Viruses that infect prokaryotes (phages) are key components of microbial communities in marine sediments, where they influence the mortality, diversity, and biogeochemical cycling of prokaryotes [1, 2]. Through lytic infection and subsequent cell lysis, phages promote the release of bioavailable organic matter, thereby contributing to the recycling of carbon and nutrients [3]. In a cross-oceanic study [4], the relative importance of phages in prokaryote mortality and carbon cycling was shown to increase with water depth as the flux of labile organic matter by sinking particles decrease. At water depths >1000 m, phages were estimated to account for ~90% of prokaryotic mortality in marine sediments, sustaining up to 35% of the bacterial carbon demand [4]. These early values may not be representative for the deep ocean in general [5, 6], but they reflect the potential importance of phages for benthic biogeochemical cycling.

In addition to the lysis-driven effects of phages on biogeochemical cycling, phage-encoded auxiliary metabolic genes (AMGs) influence the metabolic function of their host. AMGs are found in both lytic and temperate phages and they have the potential to be utilized at any stage of host infection. Some of the most studied examples (e.g. photosynthesis [7], sulfur oxidation [8], and nucleotide biosynthesis [9]) are AMGs in lytic phages, which contribute to or alter key host metabolic functions to optimize viral proliferation during infection. Although viral reprogramming of host metabolic machinery through AMGs during the lytic infection phase has been studied in several virus-host systems, the role of AMGs in temperate phages during the lysogenic phase is less known.

Temperate phages spend part of their life cycle as prophages lying dormant inside their hosts, and it has been suggested that their AMGs increase host fitness rather than that of the phage itself [10, 11]. This has led to the suggestion that phage life cycle strategies and host habitat select for the role and composition of AMGs in marine phages [12]. One example is AMGs encoding virulence factors (e.g. [10, 13, 14]), which have been experimentally demonstrated to increase host virulence. However, the majority of studies (e.g. [12, 15]) into the potential role of AMGs involved with metabolic function are entirely based on *in silico* identification and annotation of functional genes in prophage genomes, without experimental evidence to support gene function prediction and gene use. Therefore, studying AMG utilization in temperate phage-host systems experimentally is important for validating *in silico* predictions and obtaining insights into the function and regulation of AMGs and how they influence host fitness and ecology.

Although prophages represent a hereditary biosynthetic burden and a latent genetic bomb, it has been suggested that efficient dispersal of phages combined with mutualistic interactions between temperate phages and their hosts contributes to the stable coexistence of free phages, prophages, and hosts [16]. Furthermore, environmental conditions also seem to play an important role in the overall prevalence of lysogenized populations [17]. Little is known about the prophage induction frequency and regulation, especially in environmental systems. Known prophage induction signals have traditionally been associated with host DNA damage, activation of the SOS response, and poor metabolic state of the host cell [18–20]. More recently [21], quorum sensing signaling has been shown to regulate prophage induction in response to cell density changes, suggesting that bacterial hosts may take control of the lysis-lysogeny switch to promote host fitness at high cell densities, further emphasizing the potential of prophages to provide functional advantages to their host. Therefore, both the direct effects of prophage manipulation of host genetics and the indirect effects of viral-induced cell lysis in the marine environment may play an important role in catalyzing biogeochemical cycling on a global scale.

Hadal trenches (>6000 m depths) represent the deepest parts of the global ocean. These extreme environments have been shown to be hotspots of microbial activity driven by seismic-related mass-wasting and downslope funneling of organic matter, compared with the adjacent abyssal plain sediments (4000–6000 m depths), which are characterized by low input of organic material and low microbial activity [22–24]. Recently, analyses [25–27] of microbial metagenomes from hadal sediments documented a large diversity of viruses in these environments and found indications of a variety of AMGs potentially supporting host metabolic functions, suggesting that prophages influence biogeochemical cycling in these environments [21]. However, these studies are mostly based on sequence derived data, and experimental evidence of AMGs supporting host and/or phage performance in the deep sea is limited [28, 29]. In the current study, we isolated and characterized a temperate bacteriophage and its *Pseudomonas* sp. host obtained from sediment samples collected along the axis of the Kermadec Trench at a depth of ~10 000 m, as well as from an adjacent abyssal site. The phage encodes a chitinase gene, and we showed that the prophage significantly stimulated host performance in the presence of chitin, and provided evidence to suggest that the host was able to control the lysis-lysogeny switch. We hypothesize that prophage AMGs play important roles in the functional properties and adaptations of deep-sea bacteria to variable environmental conditions, thus influencing benthic biogeochemical cycling.

## Materials and methods

### Sampling and isolation

Kermadec Trench is ~1500 km long and ~ 60 km wide, with a maximum depth of 10 047 m, and is located in a relatively oligotrophic region of the southwest Pacific [30]. We sampled two hadal sites (K4:31°08.41′S, 176°48.48′W, 9300 m and K5:31°56.14′S, 177°17.48′W, 10010 m) in the trench and one reference site at the abyssal plain adjacent to the trench (K7:32°11.22′S, 176°33.66′W, 6080 m). Sediment cores were collected by subsampling in a box corer (50 × 50 cm).

Samples were retrieved and processed as previously described [31]. In brief, samples used for bacterial isolation were collected from sediment slurries prepared with virus-free bottom water prefiltered trough a 30 kD Vivaflow 200 polyethersulfone membrane (Sartorius, Göttingen, Germany) and addition of 50 mmol L^−1^ tetrasodium pyrophosphate (Merck, Darmstadt, Germany). Triplicate samples of 1.8 ml were added sterilized molecular-grade glycerol (Sigma-Aldrich, CA, USA) to a final concentration of 15% before storage at −80°C until further use.

For bacterial isolation, 100 μl of the cryopreserved samples were spread on thiosulfate–citrate–bile salts–sucrose agar plates (Sigma-Aldrich, CA, USA), which selects for pseudomonads and vibrios, and incubated at room temperature for 3 days. A single colony morphotype appeared on all plates, and from each of the five stations and depths, ~2–3 colonies were picked and transferred to 96-well plates with Marine Broth (MB) made from 5 g Bacto Peptone (Gibco, ThermoFisher Scientific, MA, USA), 1 g yeast extract (Sigma-Aldrich, CA, USA), and 20 g sea salts (Sigma-Aldrich, CA, USA) in 1000 ml MilliQ water and autoclaved at 121°C for 20 min. A total of 96 isolates were cultured and purified three times by spreading on MB agar plates (1 L of MB amended with 15 g agar (Sigma-Aldrich, CA, USA)) incubated at room temperature, and single colonies were isolated (Table S1). Cryostocks in 15% glycerol final concentration were prepared for each isolate and stored at −80°C.

For phage isolation, 10 of the 96 bacterial isolates were selected for use in the double agar overlay method [32]. In brief, bacteria were grown overnight in MB before being diluted with MB to an OD_600_ of 0.1. Bacterial culture (300 μl) was mixed with 100 μl of 0.22 μm filtered (Qmax syringe filter; Frisenette, Denmark) cryopreserved slurry from the same sample as the bacteria were isolated from, before adding it to 4 ml molten soft agar (MB amended with 0.4% agar, melted, and kept at 43°C). The mixture was poured onto MB agar plates to produce double agar overlay plates. The plates were incubated for 3 days at room temperature and inspected for clearing zones (plaques) as an indication of phage infection in the bacterial lawn [33].

Plaques were obtained on a bacterial isolate originating from station K4 inside the Kermadec Trench [31] at 2–4 cm depth in the sediment, and this susceptible host was subsequently named KT_2_4. This species was identified as a *Pseudomonas* sp. by subsequent genomic analyses (see below). Individual plaques were obtained using a sterile pipette tip, transferred to 2 ml SM buffer (5.8 g NaCl_2_, 2 g MgSO_4_•7H_2_O, 7.5 g Tris–HCl (Trizma, pH 7.5;Sigma-Aldrich, CA, USA), and 0.1 g Gelatin (Sigma-Aldrich, CA, USA) in 1000 ml MilliQ water), and vortexed. The phage solution was then centrifuged (10 000 × *g*, 10 min) and 0.22 μm filtered (Qmax syringe filter, Frisenette, Denmark). The isolated plaques were subjected to three rounds of purification, plating a dilution series of the phage suspension using the double agar overlay method described as above. For each round a single plaque was purified and the final purified phage was named Pseudomonas phage KT1.

Susceptibility of all 96 environmental isolates to phage KT1 was tested using the small drop spot assay [34], where drops of purified phage were spotted onto lawns of the individual bacteria embedded in soft agar. In the case of growth inhibition in the spotted zone, the environmental isolate was considered susceptible to the phage. Prior to the isolation procedures, the sterility of the 0.02 μm filtered water used for the slurries was tested for bacterial and phage isolation. As no bacteria or phages were isolated from the seawater used for dilution, it was concluded that the isolates were derived from the sediment samples. The isolated bacteria (all *Pseudomonas* sp.; see below) were grown in MB or on MB agar, unless otherwise specified.

### Phage and host characteristics

#### Phage buoyant density, morphology, and life cycle characteristics

Phage buoyant density was determined using density gradient centrifugation of the phage stock in a CsCl gradient (100 000 × *g*, 60 min, Beckman Optima Ultracentrifuge, CA, USA) and subsequent collection and quantification of infective phages in 45 density fractions to identify the density layer containing the most phages.

Pseudomonas phage KT1 morphology was observed using a JEM-1010 transmission electron microscope (Jeol, Tokyo, Japan) by mounting them on negatively stained copper grids as previously described [35].

Phage kinetic parameters were determined by one-step growth experiments to measure the latency time, burst size, and adsorption rate of the phage during infection [36]. Phage adsorption rate (K) was calculated from the decrease in unabsorbed phages over time according to the following equation: K = 2/3/B*log(P0/Pt), where B is the concentration of bacteria (cells per milliliter), P0 = plaque-forming unit (PFU) at time zero, and Pt = PFU in the supernatant (i.e. phages not adsorbed) at time t (min). The adsorption rate (K) is the velocity constant (ml/min) [33]. For latency times and burst sizes, samples for PFU were collected every 10 min for 70 min and quantified using the spot assay [34].

#### DNA extraction and sequencing of pseudomonas phage_KT1 phage and KT_2_4 host and environmental isolates

Six environmental bacterial isolates, including the WT host, were selected for full genome sequencing. Single colonies were transferred to MB and incubated overnight at room temperature before DNA extraction was performed using the Wizard Genomic DNA Purification Kit (Promega, WI, USA) following the manufacturer’s instructions. DNA was sequenced on an Illumina DNBseq platform by BGI (Shenzhen, China). Whole-genome preprocessing and assembly were performed using Geneious Prime (2023.1).

For whole genome sequencing of phage KT1, the phage was proliferated in liquid cultures using the wild-type of KT-2-4. In triplicates, 1 ml phage stock was added to 25 ml bacterial cultures at an OD_600_ of 0.1 and incubated overnight at room temperature. The lysed cultures were centrifuged (10 000 × *g*, 20 min), and the supernatant were filtered (0.2 μm Sterivex, Millipore, MA, USA). The phage stocks were then concentrated using polyethylene glycol 8000 (PEG-8000, Sigma-Aldridge, CA, USA) as described in Castillo et al. [37]. The phage pellet was resuspended in 1 ml SM buffer without gelatin before DNA was extracted and sequenced as described above for the bacterial isolates. Genome annotations were done using DRAM (v0.1.2) [38] and when possible, checked for consensus with Prokka (v1.14.5) [39].

### Phage and host phylogeny

Phylogenetic trees were constructed for the isolated bacterium *Pseudomonas* sp. and phage_KT1. The reference database for the bacterium was made from 113 *Pseudomonas* genomes downloaded from NCBI (MD, USA, October, 2023) and 10 *Pseudomonas* genomes from deep-sea or hadal environments [40–44]. *Cellvibrio Japonicum* Ueda107 was used as the outgroup. The genes 16S rRNA, *rpoD*, *rpoB,* and *gyrB* were extracted from each genome and the phylogenetic tree was constructed as described Gomila et al. [45]. In brief, the phylogenetic tree was constructed on the concatenated, individual gene alignments using mafft —auto (v7.520) [46], in the order: 16S rRNA, *gyrB*, *rpoD,* and *rpoB*. A maximum-likelihood tree was generated using IQ-TREE (v2.2.2.6) [45]and visualized using FigTree (v1.4.3). For the phage phylogeny, a full genome blast search was attempted with no results. Instead, a reference set of phage genomes were compiled from all Pseudomonas phages from marine sources (NCBI, MD, USA, October 2023) together with the top 20 best hits and hits originating from environmental sources from a BLASTp search of the terminase large subunit gene from phage_KT1. The phage sequences where downloaded, reannotated using DRAM [38] and the terminase large subunit gene was extracted from each genome or contig. Alignment and tree building were done as described above.

### Distribution pf phage and host in environmental samples

#### Metagenomic sequencing and screening for Pseudomonas KT_2_4 sequences in environmental samples

To investigate the presence and relative abundance of the host organism, KT_2_4 *in situ*, a per-sample coverage was estimated using metagenomic samples generated from the original sediment samples. DNA extraction, library construction sequencing and raw read quality filtration is described in detail in Trouche et al. [47], (2023). The raw, quality checked reads were mapped onto the genome of KT_2_4 using Bowtie2 [48] and Samtools [49]. MetaBAT2 [50] was used to generate the per-sample coverage table (Table S2).

#### Quantification of phage spatial distribution in the sediment

To determine the *in situ* density of phages infective against the *Pseudomonas* sp. host in the environmental samples, a PFU assay was done using the original glycerol-amended sediment slurries diluted 10x and 100x. One hundred microliters of either dilution was plated as described above using the wild-type KT_2_4 and the double agar overlay method. PFUs were enumerated from the dilutions and used as a proxy for total abundance of infective phages in the sediment samples.

### Pressure effects on host growth, phage viability, and phage-host interactions

To quantify the effects of hydrostatic pressure on the growth of the *Pseudomonas* sp. KT_2_4 isolate, bacterial culture was inoculated in 10% MB medium at a starting density of ~10^6^ cells ml^−1^ before filling 6 ml exetainers in 10 replicates. Five replicates were incubated at atmospheric pressure (controls) or 1000 bar, respectively. At 1000 bar, the pressure corresponded to the hydrostatic pressure at 10000 m water depth. In parallel, the effects of pressure on phage_KT1 infectivity were determined at atmospheric pressure (control), 500 bar, and 1000 bar in five replicates of 3 ml exetainers containing (~10^6^ PFU ml^−1^ in SM buffer).

Pressure incubations were conducted in rotating cylindrical stainless-steel tanks (100 mm in diameter and 400 mm in length; Dustec GmbH, Wismar, Germany) ([51]. The experiment was run for 72 h at 4°C, and samples for CFU and PFU counts were collected immediately before pressurization (T_start_) and at the end (T_end_) of the experiment. Control incubations were performed at atmospheric pressure in acrylic cylinders in the dark and rotated at 5 rpm on custom-made roller tables [51].

### Infection experiments and quantification of lysogeny and host resistance

To examine phage infection characteristics and development of resistance, the wild-type KT_2_4 host was inoculated in triplicate in 100 ml MB medium at OD = 0.1 with 3 × 10^8^ PFU ml^−1^ of phage_KT1 with control cultures without phages. Samples were collected every 2 h for 12 h to measure OD_600_ and PFU. At the last time point, samples were spread on MB agar, and 100 single colonies were obtained and tested for susceptibility to Pseudomonas phage_KT1 by spot assay (Table S3).

#### Analysis of lysogenization by pseudomonas phage KT1 in experimental and environmental *pseudomonas* sp. isolates.

A subset of 83 of the isolates obtained from the infection experiment above (Table S3) was further screened for lysogenization with phage_KT1 phage analyzing for the presence of three phage_KT1 genes: A polymerase (ORF 7), a hypothetical protein (ORF 33), and a structural gene (ORF 2) (Fig. S1). DNA was extracted from all the individual isolates, and primers were designed for PCR amplification of the three phage genes (Table S4). If all three genes were present in the genomic DNA of an isolate, the bacterium was considered to be a lysogen.

This analysis was repeated on 96 isolates from the environmental samples to examine the presence and distribution of Pseudomonas phage_KT1 as a prophage in the environmental populations.

From these analyses, three different infection phenotypes of the host were identified: (i) a phage susceptible type, which did not contain the prophage (e.g. the wild-type strain KT_2_4 (WT)), (ii) a lysogenic phenotype (R+) resistant to phage infection because it carried the phage_KT1 as a prophage, and (iii) a resistant phenotype (R-) with no prophage, which had acquired resistance in some other way. From here on, the three infection phenotypes are referred to as WT, R+, and R-, respectively.

Genome annotation of Pseudomonas phage_KT1 revealed an AMG encoding a putative chitinase, which was annotated as a different gene than the chitinase gene found in the host genome. Therefore, a series of experiments was designed to test and quantify the extent to which this chitinase gene encoded a functional protein, its metabolic implications in the lysogens, as well as the general implications of phage resistance. For these experiments, we selected representative clones for each of the three infection phenotypes of the *Pseudomonas* sp. KT_2_4 (i.e. WT, R+ and R-). Activity, growth rate, and biofilm formation of these three strains were quantified under different substrate conditions, with and without chitin.

### Effects of lysogeny on host cell performance

#### Chitinase activity

Chitinase activity of the three strains was quantified using both a fluorogenic assay in a 96-well plate with a chitin derivative as the substrate [52] and a plate assay with chitin-enriched agar. For the fluorogenic assay, the bacterial strains were grown to exponential phase (OD_600_ = 0.5–0.55) in 1% MB medium enriched with 0.01% chitin (Sigma-Aldrich, CA, USA), and then incubated with 5 μM (final concentration) of the substrate methylumbelliferyl(MUF)-N-acetyl-β-D-glucosaminide (Sigma-Aldrich, CA, USA) in triplicate for 1 h using growth medium as a blank. Chitinase activity was calculated from the increase in fluorescence caused by the enzymatic degradation of the non-fluorescent MUF-N-acetyl-β-D-glucosaminide to the fluorescent MUF product during incubation, and quantified on a plate reader (FLOUstar OPTIMA, BMG Labtech GMbH, Ortenberg, Germany) at Ex_355nm_ and Em_460nm_. For the plate assay, MB plates amended with 2% final concentration of chitin and spots of 10 μl of overnight cultures (OD_600_ = 1.1) of the bacterial strains were added and incubated for 48 h at room temperature. As the embedded chitin was degraded by the chitinase secreted by the bacteria growing in the center for the plate, a clearing zone around the bacterial colony developed. Chitinase activity was measured as the diameter of the clearing zone.

#### Growth rate and net cell production

Growth rate and net cell production of the three strains were measured in the presence of chitin. Overnight cultures of the strains in 10% MB medium were inoculated into 50 ml 1% MB medium amended with 0.01% chitin in triplicate in 250 ml square bottles (Nunc, ThermoFisher Scientific, MD, USA) at a final concentration of ~1 × 10^6^ cells ml^−1^. The cultures were incubated for 60 h at 16°C, every 6–10 h, 1 ml was removed for quantification of cell density, fixed with 2% final concentration of glutaraldehyde, and stored at −80°C. For quantification of cell density on a FACS Canto II flow cytometer (Becton-Dickinson, NJ, USA), samples were diluted in TE buffer, and the cells were stained with the fluorescent SYBR-Green stain for 5 min [31]. The 60 h experiments covered the entire exponential growth curve of the strains that entered the stationary phase after ~50 h of incubation. The growth rate was calculated from the exponential increase in cell density during the initial 36 h, and net cell production was defined as the maximum cell density reached after ~55–60 h of incubation.

#### Chitinase expression levels

Expression levels of the prophage-encoded chitinase gene in *Pseudomonas* sp. KT_2_4 (R+) was evaluated by RT–qPCR. Overnight cultures and *Pseudomonas* sp. KT_2_4 (R+) in 10% MB was inoculated into 60 ml 1% MB in 250 ml square bottles (Nunc) with and without 0.01% chitin supplementation. *Pseudomonas* sp. KT_2_4 (WT) was analyzed in parallel as a negative control. The experiment was done in triplicates, resulting in 12 bottles in total. The cultures were incubated for 5 h, allowing the bacteria to enter exponential stage of growth. Samples of 20 ml were taken from each replicate, followed by centrifugation at 2500 g for 10 min. The supernatant was discarded, and the bacterial pellet was used for total RNA extraction, using PureLink RNA Mini Kit (Invitrogen, MA, USA) following the manufacturer’s instructions. Before eluting RNA from the column, DNae treatment (Invitrogen, MA, USA) was performed twice. The final RNA was measured by Qubit™ RNA High Sensitivity (HS) method, according to manufacturer’s protocol (Invitrogen, MA, USA).

Reverse transcription of the RNA was performed using PrimeScript RT Master Mix (Takara Bio Inc., Tokyo, Japan) after adjusting the amount of total RNA to the same level in each reaction, following manufacturer’s protocol. The obtained cDNA was used to evaluate the expression levels of chitinase in the presence and absence of chitin for the two strains by quantitative PCR, using TB Green Premix Ex *Taq* II *(Takara Bio Inc, Tokyo, Japan). Two sets of primers targeting the chitinase gene of *Pseudomonas* phage_KT1 were designed: chi_01F (5’-TTACCGGACGTGCGAACTAT-3′) and chi_01R (5’-CCGTTGATGCGCTTAGTGAT-3′), chi_02F (5’-GTCGATCACTGAGCAGCAAC-3′), and chi_02R (5’-CACGCACATAACGCAATTGG-3′). Two housekeeping (HK) genes (*recA* and *anr*) that have been used in a previous study to normalize mRNA from *Pseudomonas* sp. strains in qRT-PCR were used [53]. Specific primers targeting the HK genes of *Pseudomonas* sp. KT_2_4 were designed as follows: recA_01F (5’-GAGGTTGTCGGTAGCGAAAC-3′) and recA_01R (5’-GATCTTGCTGCCGTTGTAGG-3′), anr_01F (5’-AGCAAATCACCGGTTTCCAC-3′), and anr_01R (5’-GGTTTTCTTCGACAGCAGCA-3′), respectively. The PCR mixture for each reaction was prepared as follows: TB Green Premix Ex *Taq* II 12.5 μl, Forward Primer (10 μM) 1 μl, Reverse Primer (10 μM) 1 μl, nuclease-free purified water 8 μl, RT cDNA mix (sample) 2.5 μl. The qPCR amplifications were performed in hard-shelled PCR low-profile, semi-skirted plates (BioRad, CA, USA) covered with an adhesive plate sealing film (BioRad, CA, USA) using a Bio-Rad CFX Connect (BioRad, CA, USA), following a 2-step PCR protocol including melting curve.

#### Biofilm formation

Biofilm-forming properties of the strains were quantified using the crystal violet method [54] and modified to 96-well plates. Twenty microliters of overnight cultures in MB were inoculated into 200 μl of fresh MB in eight replicates, and the plates were incubated for 24 h at room temperature. After staining with crystal violet, washing, and detachment of the stain using 96% ethanol, the absorbance was measured at Abs_595nm_ in the plate reader. A parallel experiment in which the WT strain was incubated with phage_KT1 for 24 h prior to biofilm measurement was performed to examine the role of phage infection in biofilm formation.

### Effect of substrate composition on prophage induction

To examine the effect of substrate composition on prophage induction dynamics, changes in the induction of prophages from the lysogenic strain R(+) were quantified in response to medium strength and chitin availability in the medium. The strain was cultured in a concentration series of MB covering 0.1%, 1%, 10%, and 100% medium strength, with and without 0.01% chitin. Growth was monitored by CFU counts, and the induction of prophages was quantified by PFU as a proxy for the concentration of viable phage_KT1 obtained as the phage density leveled out when the culture reached the stationary phase.

## Results

### Characterization of the phage-host system

The phage isolate had a siphoviridae-like morphology with a buoyant density value of 1.44 g ml^−1^ and a size of 140 nm (Fig. S2). Life cycle characteristics showed an adsorption rate of 9.712 x10^−10^ ± 1.28 *10^−10^ ml min^−1^, a latent period of 40 min and a burst size of 4.5 ± 2.0 phages cell^−1^ in MB medium at 20°C (Fig. S2).

The five environmental bacterial isolates and phage host KT_2_4 were identified as belonging to the genus *Pseudomonas* based on the 16S rRNA gene. To confirm their position within the *Pseudomonas* group, an additional multisequence alignment analysis was done. The five environmental isolates appeared to be the same strain of *Pseudomonas* sp. and were closely related, but not identical, to the host KT_2_4. All six *Pseudomonas* isolates from the present study were placed within a subgroup under *Pseudomonas fluorescens* [55]. The isolates in this subgroup were primarily associated with the soil and the rhizosphere. Furthermore, none of the *Pseudomonas* genome sequences in this study clustered closely with previously reported *Pseudomonas* strains found in deep-sea environments (Fig. S3). The isolated phage formed a sub-cluster with three other phages. The closest relative was an undescribed prophage found in *P. fluorescens* strain Ps 77 (GenBank: LCYB01000031). A temperate Marinobacter phage infecting algae-associated bacteria [56] and a metagenome-assembled viral bin identified as a *Caudoviricetes* (GenBank: BK029073) were the other two members in the subcluster (Fig. S4).

Ninety-six *Pseudomonas* isolates obtained from the environmental samples were screened for lysogenization with phage_KT1 phage based on the presence of three phage_KT1 genes (Table S1). Of these, the majority (95%) were PCR-positive for all three phage genes, and thus contained phage_KT_2_4 as a prophage and were resistant to re-infection. Of the remaining 5% (4 isolates) that were non-lysogenic, only two were susceptible to infection by Pseudomonas phage_KT1.

Genome sequencing of the original bacterial isolate KT_2_4, used as a host strain for isolation of the phage showed a 6 103 812 bp genome bacterium belonging to the *Pseudomonas* genus. The genome had 60% GC content and contained five putative prophage genomes and seven questionable CRISPR arrays.

Whole genome sequencing of phage_KT1 revealed a 40 631 bp genome with 52 open reading frames and a GC content of 57.7% (Fig. S1, Table S5).

The genomes of the host KT_2_4 and phage_KT1 each encoded a putative chitinase gene. The bacterial chitinase gene is 1065 bases long and translates to 354 amino acids. The chitinase gene was annotated as glycosyl hydrolase in the GH18 family, which is a group of chitinases catalyzing random endo-hydrolysis of the (1- > 4)-beta-linkages in chitin and chitodextin compounds. In the bacterial genome, the chitinase is preceded by lytic polysaccharide monooxygenase (LPMO), a group of proteins that oxidizes the cleavage of glycosidic bonds. Downstream of the chitinase a gene encoding a potential repressor similar to the GntR transcriptional regulators. Besides this, most of the surrounding genes of the chitinase are hypothetical proteins with unknown function. The putative chitinase gene sequence of phage_KT1 differs from that of the bacterial chitinase gene and is much smaller than its bacterial counterpart, with only 534 bases and 177 amino acids. The phage gene was annotated as a chitinase belonging to the GH19 family.

### Distribution of the *Pseudomonas* KT_2_4 and infective phages in Kermadec Trench sediments

**Figure 1:**
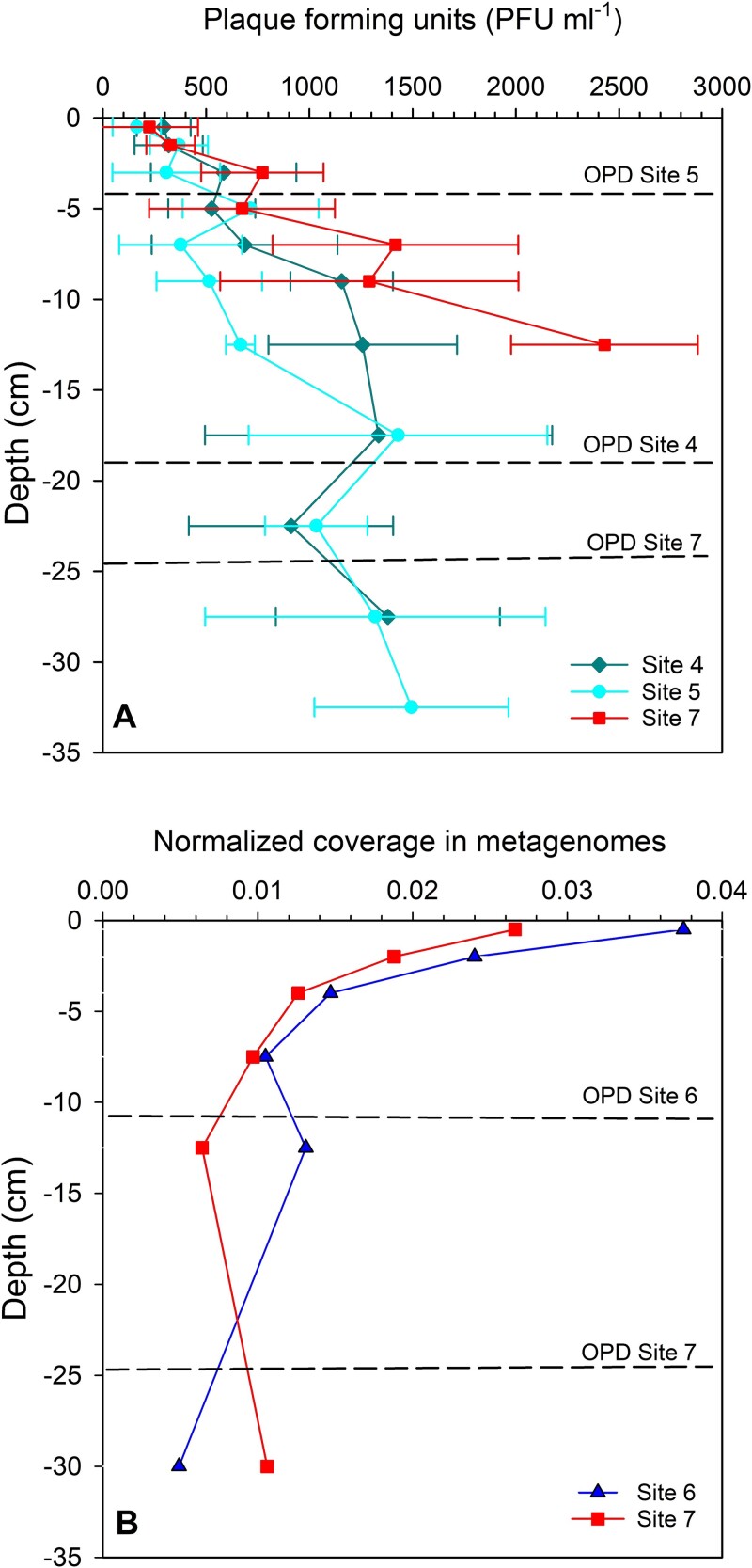
(A) Distribution of infective phages (PFU) in the sediments at the hadal Sites 4 and 5 and the abyssal Site 7. (B) Distribution of the relative abundance of *Pseudomonas* sp. KT_2_4 genomes in sediments obtained from the metagenomes of the samples from the hadal Site 6 and abyssal Site 7 horizontal lines indicate the oxygen penetration depths at the three sites (from Glud et al 2021 [22]).

The *in situ* abundance of the phage infective units (Fig. 1A) showed the presence of the phage both inside the hadal trench (Sites 4 and 5) and in sediment from the abyssal plain (Site 7). Furthermore, viable phages were found at all the sampled depths (0–33 cm). At all sites, there was a gradual increase in viral density with sediment depth, from 163 ± 117 to 294 ± 1135 PFU ml sediment^−1^ in surface sediment to a maximum of 1380 ± 543 PFU ml sediment^−1^ and 1495 ± 470 PFU ml^−1^ at 25–35 cm depth at hadal Sites 4 and 5, respectively. In sediment from the abyssal plain, phage abundance reached 2430 ± 1352 PFU ml sediment^−1^ at a depth of 15–20 cm.

In the metagenomic data, the relative coverage of Pseudomonas KT_2_4 suggested that it was most abundant in the surface sediment and decreased gradually with depth to 5–10 cm where some variation was observed between hadal and abyssal sediment at 15–30 cm depth (Fig. 1B).

### Effects of pressure on phage and host stability and interactions

To investigate the effects of hydrostatic pressure, we exposed the phage and the bacterial host to different pressures, both individually and in combination. In the absence of the host, the infective phage density remained relatively constant for the duration of the experiment at different pressures (Fig. S5), varying from 1.2 × 10^6^ PFU ml^−1^ at T = 0 to 2.1 × 10^6^ ± 6.2 × 10^5^ PFU ml^−1^ at atmospheric pressure, 1.7 × 10^6^ ± 5.3 × 10^5^ PFU ml^−1^ at 500 bar and 1.8 x10^6^ ± 4.1 × 10^5^ PFU ml^−1^ at 1000 bar with no significant effects of pressure on phage infectivity after 68 h of incubation (*P* > .05, Fig. S5).

The bacterial incubations were performed at atmospheric pressure and 1000 bar pressure, and here there was also no significant (t-test, *P* > .05) effect of pressure on cell growth during the incubation as the cell density increased from 1.0 × 10^6^ ± 5.9 × 10^5^ CFU ml^−1^ at T = 0 to ~1 × 10^7^ CFU ml^−1^ at both atmospheric pressure and 1000 bar at the end of the incubation (Fig. S5).

The combined phage-host experiment was also conducted at atmospheric pressure and 1000 bar pressure, and showed similar results, with no significant effect (t-test, *P* > .05) of pressure on phage production. During the incubation, phage production increased from ~3 × 10^3^ phages ml^−1^ at t = 0 to 1.0 and 0.8 × 10^4^ phages ml^−1^ in the atmospheric and 1000 bar incubations, respectively, after 72 h (Fig. S6). Similarly, there was no significant effect on bacterial density, which increased from 2.4 × 10^6^ cells ml^−1^ at t = 0 to 2.4 × 10^7^ ± 1.1 × 10^7^ bacteria ml^−1^ and 2.1 × 10^7^ ± 1.2 × 10^6^ bacteria ml^−1^ at atmospheric and 1000 bar pressure, respectively after 72 h incubation (Fig. S6). Overall, these experiments suggest that both host and phage are piezotolerant.

### Phage-host interactions and selection for lysogenization

**Figure 2:**
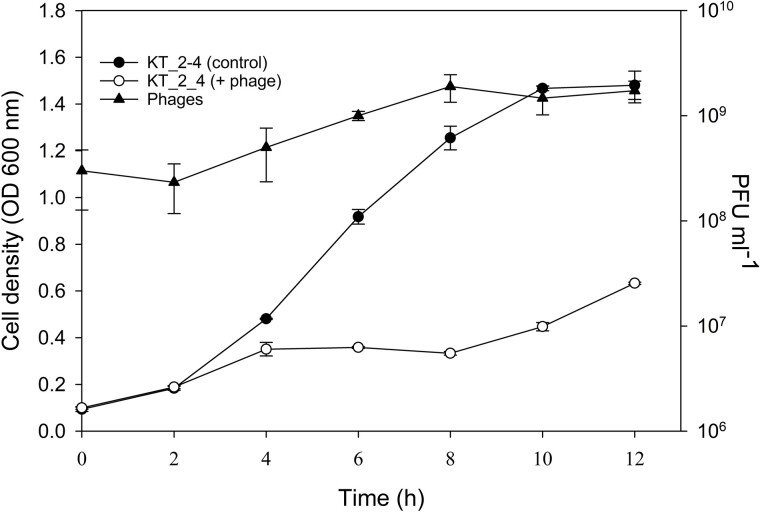
Growth of *Pseudomonas* sp. KT_2_4 WT host in the presence of Pseudomonas phage_KT1 and control cultures without the phage, along with phage production.

When the phage-sensitive wild type strain KT_2_4 was inoculated at OD = 0.1 with 3 × 10^8^ PFU ml^−1^ of Pseudomonas phage_KT1, there was a strong controlling effect of the phage on bacterial growth relative to control cultures without phage during 12 h incubation. The control cultures grew exponentially and reached a stationary phase at an OD of 1.5, whereas the phage-exposed culture remained at an OD of ~0.35 until 8 h, and then increased to 0.63 after 12 h. The production of 1.4 × 10^9^ phages ml^−1^ thus reduced the bacterial cell density to 43% of the control density (Fig. 2).

Of the 100 clones isolated from the phage-exposed cultures after 12 h, 61 clones were resistant to phage infection and the rest showed some variation in sensitivity to infection (Table S3). A subset of 83 clones was further tested for lysogenization, 55% of which were lysogenized, whereas 45% did not contain the prophage. Lysogenization provided total resistance against re-infection by Pseudomonas phage_KT1, whereas only one of the non-lysogenic isolates, KT_2_4 (R −) (Table S3), was resistant to phage infection. Overall, phage exposure was selected for a mixed population of primarily phage-resistant lysogens and phage-sensitive non-lys ogens.

### Effects of lysogenization and resistance on host performance

Based on the different genotypes and phenotypes of isolates obtained from environmental samples as well as from culture experiments, we used three representative isolates for subsequent analysis of the effects of lysogeny and resistance on the performance of *Pseudomonas* sp. (Table S6), with emphasis on the potential role of the prophage-encoded chitinase gene.

**Figure 3:**
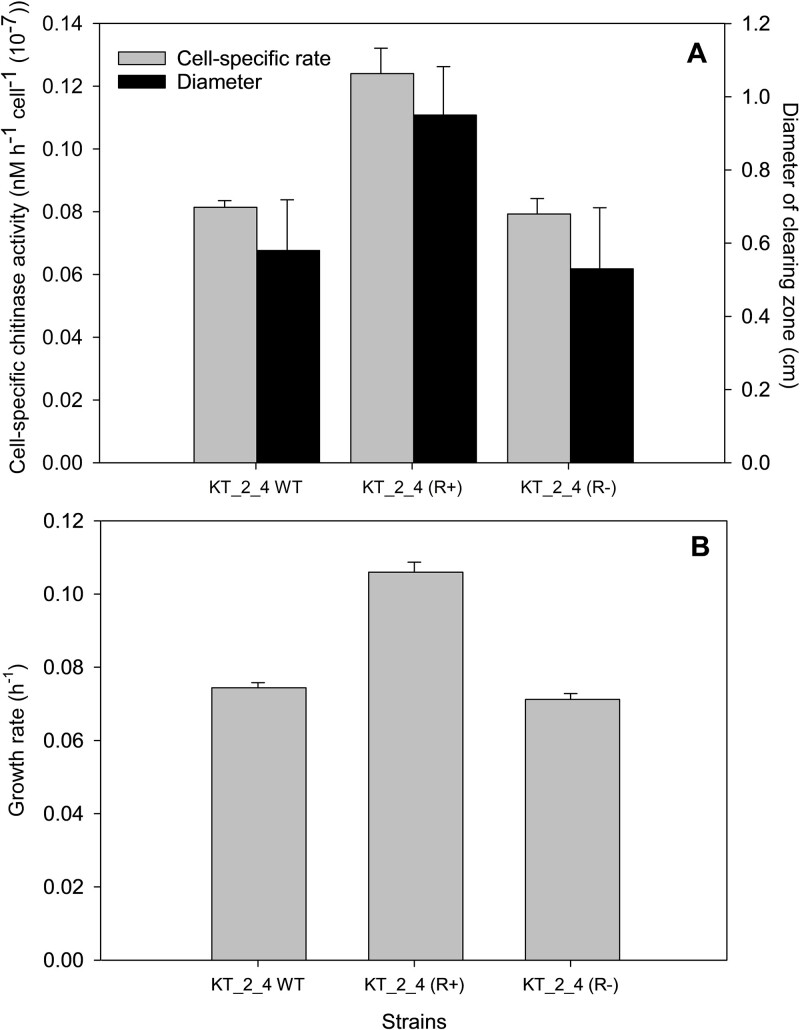
(A) Chitinase activity of the WT, R+, and R- strains measured using fluorogenic and plate assays. (B) Growth rate of the same strains during exponential growth in chitin-enriched medium in batch culture.

Quantification of chitinase activity by two independent methods demonstrated significant increases in chitinase activity in *Pseudomonas* sp. KT_2_4 (R+) relative to the non-lysogenic WT control (Fig. 3). Using the fluorogenic approach with MUF-N-acetyl-β-D-glucosaminide assay, the lysogen showed a 52% increase in chitinase activity (*P* < .001), whereas chitinase activity quantified from the enzyme-driven clearing zone in a chitin-embedded agar, showed a 64% increase relative to the WT strain (*P* < .002) (Fig. 3A). In the chitin-containing growth medium, the increased chitinase activity coincided with a significantly higher growth rate (0.106 ± 0.003 h^−1^) of the lysogenic strain than obtained for wild-type and phage-resistant strains (0.0744 ± 0.002 h^−1^ and 0.071 ± 0.002 h^−1^, respectively) (Fig. 3B; *P* < .002).

A comparison of net cell production during a 60-h experiment at 16°C for the three selected clones in media with and without 0.01% chitin supported the observed increase in the performance of the lysogenic strain KT_2_4 (R+) compared with the non-lysogenic strains (Fig. S7). In the presence of chitin, strain KT_2_4 (R+) produced 12% and 18% more cells than the non-lysogen KT_2_4 WT KT_2_4 (R-), respectively, confirming the benefit of the prophage-encoded chitinase in supporting host growth. The addition of chitin to the 1% MB medium showed that chitin significantly promoted net growth (*P* < .05) in all three strains, emphasizing that the chitinase detected in the core host genome also supported net cell growth in these incubations (Fig. S7). In the wild-type strain KT_2_4 WT, this corresponded to an 8% increase in net growth, whereas the 17% increase in growth of the lysogen in chitin-enriched medium again emphasized the importance of the prophage-encoded chitinase for chitin degradation in this *Pseudomonas* sp. (Fig. S7). The fact that the net production of the WT and lysogenic strain did not differ significantly in the absence of chitin suggests that there was no apparent fitness cost of having the prophage during growth in 1% MB medium. The lowest net production was observed in the non-lysogenic phage-resistant strain KT_2_4 (R-) in the absence of chitin, suggesting that resistance may impose a fitness cost in this medium compared with the phage-sensitive wild-type strain (Fig. S7) [57].

### Prophage-encoded chitinase expression

Quantitative RT-PCR showed a 2.3 to 3.3-fold increase in prophage-encoded chitinase gene expression in lysogenic strain KT_2_4 (R+) in 1% MB supplemented with 0.01% chitin compared to the control condition of 1% MB without chitin supplementation (Fig. S8). The WT strain KT_2_4 did not show significant amplification of this gene. Specifically, the first set of primers for the chitinase gene generated a 2.7- and 3.3-fold increase in expression compared to the control condition without chitin when calibrated with *recA* and *anr* HK genes, respectively. The second set of primers confirmed the upregulation of chitinase in the presence of chitin by 2.3- and 2.7-fold, respectively, when normalized to *recA* and *anr* (Fig. S8).

### Effects of phage and prophage on host biofilm formation

The influence of phage-host interactions on host biofilm formation was examined by quantifying the biofilm-forming properties of each individual strain as well as of the wild-type strain after exposure to the phage. The lysogenic strain KT_2_4 (R+) produced significantly (*P* < .05) more biofilm than the wild-type strain KT_2_4 (Fig. S9), and also exposure of the sensitive wild-type strain to the free phage promoted biofilm formation. The resistant non-lysogenic strain also produced less biofilm than the lysogenic strain, but the difference was not statistically significant (t-test, *P* > .05).

### Phage-host dynamics and the spontaneous induction of prophages

**Figure 4:**
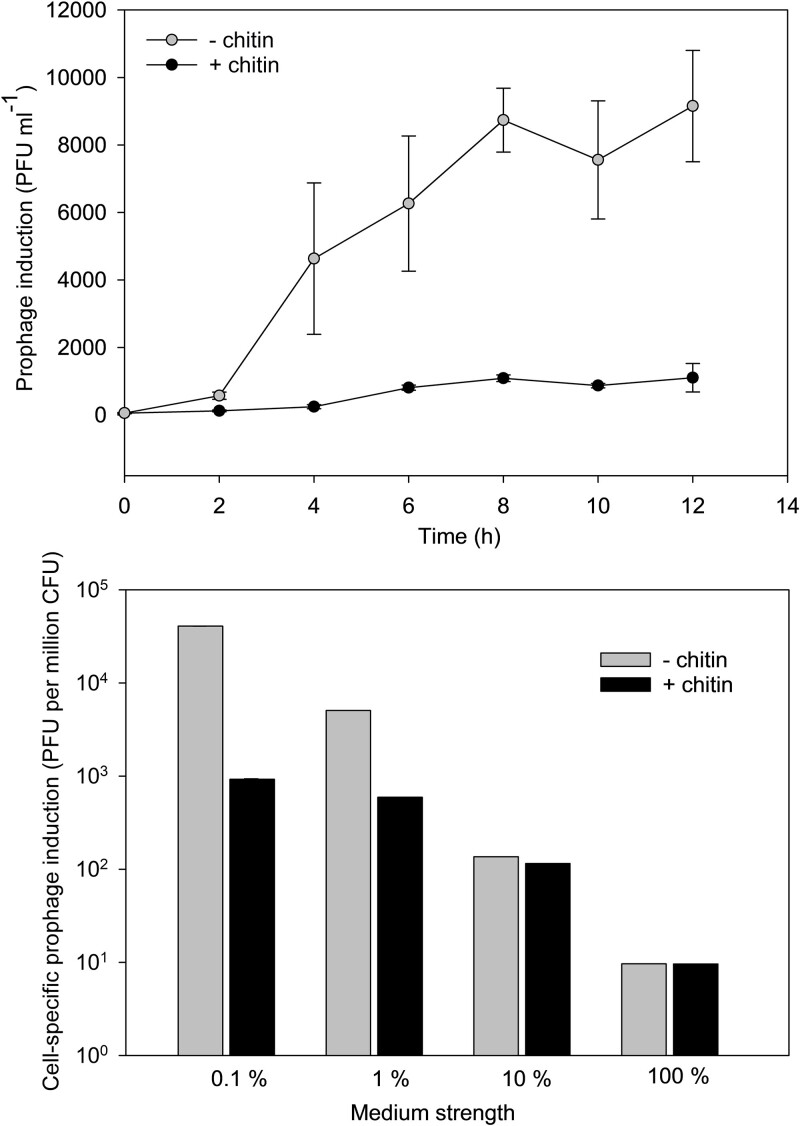
Prophage induction in *Pseudomonas* sp. KT_2_4 (R+) at various substrate conditions. (A) Release of free Pseudomonas phage_KT1 particles during 12 h growth of the lysogenic host in 1% MB medium in the presence and absence of 0.01% chitin, (B) relative prophage induction expressed as the release of free Pseudomonas phage_KT1 particles per million cells during exponential growth of lysogen at different medium strengths in the presence (black bars) and absence (white bars) of chitin.

Spontaneous induction of Pseudomonas Phage_KT1 prophages correlated with substrate concentration and composition. During growth in 1% MB, the net release of free phages from the lysogenic *Pseudomonas* sp. KT_2_4 (R+) was <1200 PFU ml^−1^ during 12 h incubation in the presence of chitin, whereas the net release of free phages by induction reached 9000 PFU ml in the absence of chitin (Fig. 4A). Comparing the prophage induction across different media strengths showed a 10-fold decrease in the concentration of free phages from ~3.5 × 10^5^ PFU ml^−1^ in 0.1% MB to ~3.5 × 10^4^ PFU ml^−1^ in 100% MB medium after 6 h of incubation. This corresponded to a decrease in cell-specific prophage induction of ~4.0 x10^4^ PFU million cells^−1^ in 0.1% MB to ~10 PFU million cells^−1^ in 100% MB, with a gradual decrease in cell-specific induction with an increase in medium strength (Fig. 4B). Combining the effects of medium strength and chitin amendment further confirmed that the medium composition affected prophage induction, as indicated by the 44-fold decrease in prophage induction upon the addition of chitin in 0.1% MB (Fig. 4B). This effect of chitin amendment on prophage induction was reduced with increasing medium strength, and at high MB concentrations (>10%), chitin addition had no effect on spontaneous induction rates (Fig. 4B).

## Discussion

This study describes a widespread, piezotolerant *Pseudomonas* sp. phage-host system isolated from one of the deepest benthic sites in the global ocean and demonstrates a substrate-controlled interaction between the *Pseudomonas* host KT_2_4 and its Pseudomonas phage_KT1, which increases the fitness of both, suggesting a mutualistic co-existence of phage and host as an adaptation to the environment they occupy.

### Characterization and distribution of *pseudomonas* KT_2_4 and pseudomonas phage_KT1

Pseudomonads are commonly found among culturable bacteria isolated on rich growth media from marine environments, reflecting their wide distribution and ability to respond quickly to nutrient input. Early reports of bacterial isolation from hadal trenches described a new species, *Pseudomonas bathycetes*, obtained from both Philippine Trench and Mariana Trench sediments at >9000 m depth [58]. More recently, several other species of *Pseudomonas* have been isolated from both sediments and deep waters of the Mariana Trench [40–43]. In addition, a temperate bacteriophage (vB_PstS-1) was identified upon induction by a *Pseudomonas stutzeri* 1–1-1b host isolated from a water sample collected at 7000 m depth in the Japan Trench [28]. However, the current isolate from the Kermadec Trench has little similarity to other deep-sea *Pseudomonas* isolates, which are also distributed broadly across the phylogenetic tree. Similarly, phage KT1 did not cluster with other deep-sea phages, suggesting a wide distribution of Pseudomonas phages across different habitats and spatial scales. The current isolation of *Pseudomonas* sp. and phage from Kermadec trench sediments is therefore in line with previous reports on the dominance of pseudomonads in culturable communities from deep-sea samples. The presence of both *Pseudomonas* KT_2_4 host and Pseudomonas phage_KT1 at all three sites, covering a horizontal distance of ~100 km, a difference in water depth from 6000 m to 10 000 m, and a vertical profile in the sediment from to 0–33 cm, suggests that this phage host system is well adapted to the different environmental conditions that prevail in these habitats [22, 57, 59]. This is also supported by the small genomic variability among the sequenced isolates of both phage and host, which suggests efficient dispersal of these phylotypes and/or limited selection for diversification, leading to a stable genomic composition of these microorganisms on a kilometer scale. Similarly, the large majority (98%) of lysogens among the environmental isolates suggested a high dispersal efficiency of the phage and co-existence with a relatively small sub-population of phage-sensitive strains that ensures re-infection and production of new phages.

The increase in infective phages with sediment depth at all stations suggested either increased prophage induction or perhaps a slower decay of infective phages in the deeper layers. The oxygen conditions at the three sites varied considerably, with oxygen penetration depths ranging from 4 cm at Site 4 to 21 cm at Site 7 [22], emphasizing that the Pseudomonas host and phage are produced and viable under both oxic and anoxic conditions. The majority of Pseudomonas species are facultative anaerobes and capable of using nitrite or nitrate as terminal electron acceptor [60], supporting the ubiquitous distribution of this phage-host system in hadal sediments and its tolerance to various environmental conditions. The observed densities ranging from 200 to 2600 PFU ml sediment^−1^ demonstrate that Pseudomonas phage_KT1 only represents a minor fraction of the total viral community of 10^8^–10^9^ viruses ml^−1^ [31], which is in line with the general finding of very high viral diversity in these environments [25].

Analysis of the metagenomic datasets from Sites 4 and 5 confirmed the presence of the Pseudomonas KT_2_4 host strain in all the samples down to >30 cm in the sediment. However, in contrast to the vertical distribution of infective phages, the relative coverage of the *Pseudomonas* host in the metagenomes suggested that the population was most abundant in the surface sediment and decreased gradually with depth to 5–10 cm. This apparent inverse relationship between the distribution of phage_KT1 and its host suggested a phage-driven reduction in host densities in the surface sediments, where the growth rates of *Pseudomonas* are expected to be highest. However, other factors are likely involved in shaping the vertical distribution of phage and host.

The wide distribution of viable phages and hosts in these deep-sea environmental conditions was also supported by the tolerance to pressurization by both *Pseudomonas* sp. and phage, as cultivability and infectivity, respectively, were unaffected by pressures of up to 1000 bar. Piezotolerant properties were also detected in *P. stutzeri* 1–1-1b from the Japan trench, which were able to grow at hydrostatic pressures ranging from 0.1–70 MPa [28].

The wide distribution of Pseudomonas phage_KT1 in and adjacent to the Kermadec Trench is in line with recent observations of the Halomonas phage vB_HmeY_H4907, isolated from surface sediment at 8900 m depth in the Mariana trench [29]. The mining of global viral metagenomic datasets for the presence of sequence homology to phage vB_HmeY_H4907 led to the discovery of a global distribution of the Halomonas phage covering bathypelagic, temperate, and tropical epipelagic and mesopelagic sampling sites. Our observations therefore support previous results and add to the increasing evidence that culturable Gram-negative Gammaproteobacteria, such as halobacteria and pseudomonads, are generalists that can grow and interact with their phages across the large temperature and pressure ranges from the surface ocean to the deep sea and from the aerobic water column to anoxic sediments.

The observed tolerance of both phage and host to exposure to the extreme hydrostatic pressure of 1000 bars demonstrated that this phage-host system can thrive under all pressure conditions in the ocean and emphasizes the versatile nature of pseudomonads. There are only a few studies on the pressure effects of phages and phage-host interactions, and the current results are, to our knowledge, the first to demonstrate phage infection and the production of progeny phages under deep-sea pressure conditions. Previous work [61] on surface water cyano- and roseophages exposed to long-term hydrostatic pressure (2 y, 30 MPa) showed variable effects on phage stability and infectivity, likely related to capsid size, volume, and shape [61]. Phages with larger icosahedral capsids were completely disrupted by pressure, whereas *Siphoviridae* phages with smaller icosahedral capsids (<60 nm) were relatively stable and maintained intact structures and infectivity [61]. Therefore, phages might require specific structural properties to maintain infectivity at hadal depths.

### Effects of the prophage on growth and chitinase activity of *Pseudomonas* KT_2_4 (R+)

When the non-lysogenic wild-type strain KT_2_4 was exposed to phage infection, the majority of the phage-susceptible population was killed, driving a strong selection for resistant clones, most of which were lysogenized, and only a minority had become resistant by other mechanisms, probably mutational changes. Representatives of these three genotypes (KT_2_4 WT: sensitive non-lysogen, KT_2_4 (R+): resistant lysogen, and KT_2_4 (R-): resistant non-lysogen) showed significant differences in growth characteristics.

The experimental comparison of growth and chitinase activity of the non-lysogen strain KT_2_4 WT and the lysogenized derivative KT_2_4 (R+) showed systematic positive effects of harboring the prophage in the lysogens when grown on a chitin-amended medium. This was not only directly reflected in the 1.5- fold higher cell-specific chitinase activity in KT_2_4 (R+) relative to the non-lysogen KT_2_4 WT, but also translated into 1.4- and 1.1-fold higher growth rates and net cell accumulation, respectively, in batch cultures. A 2.2- to 3.3-fold increase in prophage-encoded chitinase expression during exponential growth in the presence of chitin relative to conditions without chitin confirmed that the prophage chitinase gene contributed to host chitinase production. The fact that the induction of prophage induction was almost eliminated in the presence of chitin during exponential growth, further confirmed that the observed upregulation of the prophage-encoded chitinase gene in chitin-supplemented MB was associated with its function as an auxiliary metabolic gene inside the host genome.

The presence of the prophage-encoded chitinase thus improved the host’s ability to degrade chitin and significantly boosted growth performance. Chitin is a major component of crustacean exoskeletons, one of the most abundant polysaccharides in the ocean, and an important source of carbon and nitrogen [62].Therefore, the capacity to utilize chitin is a major advantage for lysogens in chitin-rich environments. In contrast, the observed decrease in cell-specific chitinase activity with medium strength suggested that the expression of chitinase is regulated by the availability of other substrates and indicated that *Pseudomonas* KT_2_4 downregulated chitinase excretion if other more labile substrates are available. The data confirmed that these chitinases are indeed functionally hydrolytic enzymes used to acquire substrate for growth in a process that is strongly regulated by the presence of chitin and general substrate availability.

As expected, the phage-resistant non-lysogenic strain KT_2_4 (R-), derived from the sensitive wild type upon phage exposure, did not show any difference in chitinase activity or overall growth rate. However, the reduced net cell production relative to wild-type KT_2_4 suggested a fitness cost related to phage-resistance mutations, as previously observed in connection with phage resistance in marine bacteria [63].

In addition to increased chitinase activity, the lysogenic strain KT_2_4 (R+) also formed more biofilms than the non-lysogens KT_2_4 WT and KT_2_4 (R-). The positive effects of prophages on biofilm formation have been observed in several lysogens [13, 21, 64, 65] and are most likely related to the release of extracellular DNA and other lysate products released upon prophage induction, which contributes to biofilm stability and structure. The observed stimulation of biofilm formation by exposing the phage susceptible strain KT_2_4 WT to the phage has also been found in marine phage-*Vibrio* interactions and has been suggested as a phage defense mechanism, as the biofilm matrix offers protection against phage infection for bacteria embedded in the biofilm [13, 66].

### Regulation of prophage induction

A recent demonstration that the lysis-lysogeny switch of some phages is regulated by host quorum sensing (QS) signaling (e.g. [21]) and a phage-encoded QS-like signal system [67] illustrated that prophage induction is modulated by a variety of environmental factors related to cell- and phage-density-dependent signal molecules.

A 4000-fold decrease in the prophage induction rate with increasing nutrient concentration from 0.1% to 100% MB medium supported the previous demonstration that high cell density favors lysogeny [21]. However, the observed influence of chitin amendment on prophage induction strongly indicated that substrate composition works as an additional environmental factor contributing to the regulation of the lysis-lysogeny switch by repressing prophage induction. Together, these observations imply that the interaction between the host and the prophage is regulated to optimize the utilization of the prophage-encoded chitinase under chitin-enriched conditions, whereas prophage release is favored under low cell density and low chitin conditions. The results also indicated that, at high nutrient concentrations and a decrease in the relative importance of chitin for supporting cell growth, the regulatory effect of chitin on prophage induction disappeared.

Overall, we suggest that prophage induction is regulated by various environmental conditions that select for either repression or activation of induction, allowing the coexistence of phages and hosts. For the host, the trade-offs between the benefit of prophage-driven chitinase activity and phage resistance on the one hand, and the cost of mortality by prophage induction (lysogen) and lytic infection (non-lysogen) on the other hand, thus seem to promote the co-existence of the lytic and lysogenic genotypes. From the perspective of temperate phages, the prophage-encoded enhancement of host fitness, protection of the phage genome inside the host genome, and occasional lytic infection securing renewal and dispersal of the phage population are also factors that contribute to explaining the phage-host co-existence and widespread occurrence in these deep-sea sediments.

### Ecological implications

**Figure 5:**
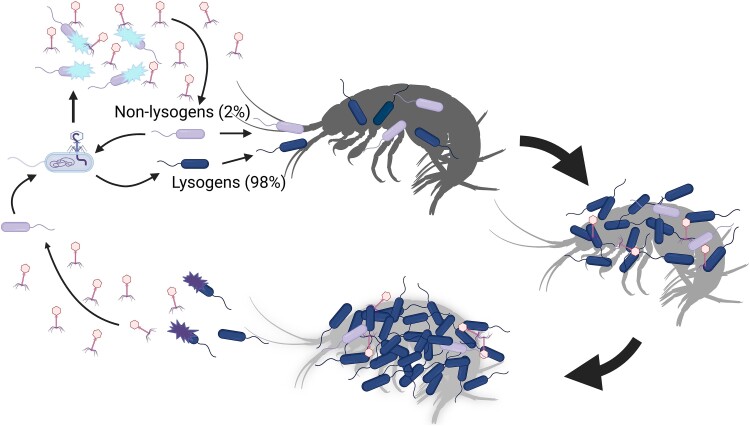
Conceptual model of the potential interaction between chitinase-encoding Pseudomonas phageKT1 and its *Pseudomonas* sp. host in deep-sea sediments in the presence of a chitin source such as an amphipod carcass. Prophage-containing lysogens (dark cells) comprise the majority of the Pseudomonas population and are favored upon colonization of the chitin skeleton. Consequently, lysogens dominate the colonizing community. Prophage induction further stabilizes bacterial biofilms and increases the chitin degradation efficiency. As the chitin source is being used and bacteria are released from the biofilm, the prophage induction rate increases, and free phages are released. A small subpopulation of phage-susceptible non-lysogens (light cells) is infected and lysed, thus maintaining the production of new phages, which ensures lysogenization of new hosts and dissemination of the AMG within the *Pseudomonas* population (made in BioRender).

The accumulation of *in silico*-based evidence that phage-encoded AMGs play important roles as modulators of host metabolic functions [68, 69] emphasizes the importance of using model systems for to quantify phage-encoded contributions to bacterial biogeochemical cycling in marine environments [70]. We propose to distinguish between AMGs as being either collaborative or selfish AMGs. Selfish AMGs are primarily encoded by lytic phages, where the AMG confers a direct advantage to the phage during proliferation by supporting key cellular functions of the host to maximize viral production. This has been observed for the *psbA* and *psbD* genes that are activated during phage infection of the cyanobacteria *Synechococcus* sp. WH7803 [71]. In contrast, collaborative AMGs can be found in lysogenic phages and provide an advantage to both the host and phage. In this case, we demonstrated a prophage-encoded chitinase utilized by the host during cellular growth, which provided a metabolic advantage to the host and prophage, and potentially also improved cellular conditions for the prophage when entering the lytic phase. Increased biofilm formation in the lysogen (R+) has been observed in association with prophage induction in other *Pseudomonas* and *Vibrio* species [65], and demonstrates that the prophage also confers other traits to its host, which are beneficial during surface colonization. Furthermore, the inhibition of phage induction in the presence of chitin suggests that the host can manipulate the lifecycle of the prophage, adding to the collaborative properties of this AMG. The collaborative nature of the interaction likely allows the host to adapt to environmental conditions, and we propose that in chitin-enriched environments, such as amphipod carcasses and exoskeletons, there is a selection for lysogens that promote biofilm formation and chitin degradation. At low chitin concentrations, the induction rate increases, allowing lytic infection of the smaller WT subpopulation (~2% of the total population) (Fig. 5), which secures the recruitment of new phage particles and lysogenization of new host cells.

Overall, the present description of a collaborative interaction between a prophage and its host, combined with previous indications of a dominance of temperate phages and a large representation of AMGs extracted from metagenomes of deep-sea benthic environments [25], suggests that prophage-encoded metabolic functional genes play an important role in host biogeochemical cycling. Recent findings of high genomic diversity in hadal sediments and indications of carbohydrate degradation capacity as an important driver in structuring the microbial communities [5], further emphasize the potential importance of phage-based dissemination of these traits. Our study provides experimental evidence that phage-bacteria interactions can affect bacterial metabolism through the regulation of prophage induction and integration, and we suggest that the variable organic matter composition in marine sediments selects for specialized and mutualistic phage-bacteria interactions and prophage-driven adaptations to variable environmental conditions. As this is a single-model system from a complex habitat, more studies are required to link the observed potential of prophage-encoded AMGs from metagenomics with an understanding of their quantitative role and regulation in benthic biogeochemical cycling.

## Supplementary Material

Middelboe_et_al_suppl_mat_Submission_ISME_rev_09012025_wraf004

## Data Availability

The genome sequence raw data are deposited at DDBJ/ENA/GenBank with accession numbers PQ316073 (Bacteriophage_KT1), CP166962 (*Pseudomonas* KT_2_4 WT), JBJGCQ000000000 (*Pseudomonas* sp. KT_Env-2), JBJGCR000000000 (*Pseudomonas* sp. KT_Env-10), JBJGCS000000000 (*Pseudomonas* sp. KT_Env-17), and JBJGCT000000000(*Pseudomonas* sp. KT_Env-32).

## References

[1] Middelboe M, Glud RN, Finster K. Distribution of viruses and bacteria in relation to diagenetic activity in an estuarine sediment. *Limnol Oceanogr* 2003;48:1447–56. 10.4319/lo.2003.48.4.1447

[2] Middelboe M, Brussaard CPD. Marine viruses: key players in marine ecosystems. *Viruses* 2017;9:1–6. 10.3390/v9100302 PMC5691653 29057790

[3] Glud RN, Middelboe M. Virus and bacteria dynamics of a coastal sediment: implication for benthic carbon cycling. *Limnol Oceanogr* 2004;49:2073–81. 10.4319/lo.2004.49.6.2073

[4] Danovaro R, Dell’Anno A, Corinaldesi C et al. Major viral impact on the functioning of benthic deep-sea ecosystems. *Nature* 2008;454:1084–7. 10.1038/nature07268 18756250

[5] Schauberger C, Thamdrup B, Lemonnier C et al. Metagenome-assembled genomes of deep-sea sediments: changes in microbial functional potential lag behind redox transitions. *ISME Com* 2024;4:1–13. 10.1093/ismeco/ycad005 PMC10809760 38282644

[6] Middelboe M, Glud RN, Wenzhöfer F et al. Spatial distribution and activity of viruses in the deep-sea sediments of Sagami Bay, Japan. *Deep Sea Res Part I: Oceanogr Res Pap* 2006;53:1–13. 10.1016/j.dsr.2005.09.008

[7] Mann NH, Cook A, Millard A et al. Bacterial photosynthesis genes in a virus. *Nature* 2003;424:741–2. 10.1038/424741a 12917674

[8] Anantharaman K, Duhaime MB, Breier JA et al. Sulfur oxidation genes in diverse deep-sea viruses. *Science* 2014;344:757–60. 10.1126/science.1252229 24789974

[9] Huang X, Jiao N, Zhang R. The genomic content and context of auxiliary metabolic genes in roseophages. *Environ Microbiol* 2021;23:3743–57. 10.1111/1462-2920.15412 33511765

[10] Bondy-Denomy J, Davidson AR. When a virus is not a parasite: the beneficial effects of prophages on bacterial fitness. *J Microbiol* 2014;52:235–42. 10.1007/s12275-014-4083-3 24585054

[11] Obeng N, Pratama AA, Elsas. The significance of mutualistic phages for bacterial ecology and evolution. *Trends Microbiol* 2016;24:440–9. 10.1016/j.tim.2015.12.009 26826796

[12] Luo XQ, Wang P, Li JL et al. Viral community-wide auxiliary metabolic genes differ by lifestyles, habitats, and hosts. *Microbiome* 2022;10:190. 10.1186/s40168-022-01384-y 36333738 PMC9636769

[13] Kalatzis PG, Mauritzen JJ, Winther-Have CS et al. Staying below the radar: Unraveling a new family of ubiquitous “cryptic” non-tailed temperate vibriophages and implications for their bacterial hosts. *Int J Mol Sci* 2023;24:3937. 10.3390/ijms24043937 36835353 PMC9966536

[14] Lopez CA, Winter SE, Rivera-Chávez F et al. Phage-mediated acquisition of a type III secreted effector protein boosts growth of salmonella by nitrate respiration. *MBio* 2012;3:e00143–12. 10.1128/mBio.00143-12 22691391 PMC3374392

[15] Heyerhoff B, Engelen B, Bunse C. Auxiliary metabolic gene functions in pelagic and benthic viruses of the Baltic Sea. *Front Microbiol* 2022;13:863620. 10.3389/fmicb.2022.863620 35875520 PMC9301287

[16] Kalatzis PG, Rørbo N, Castillo D et al. Stumbling across the same phage: comparative genomics of widespread temperate phages infecting the fish pathogen *Vibrio anguillarum*. *Viruses* 2017;9:122. 10.3390/v9050122 28531104 PMC5454434

[17] Paul JH . Prophages in marine bacteria: dangerous molecular time bombs or the key to survival in the seas? *ISME J* 2008;2:579–89. 10.1038/ismej.2008.35 18521076

[18] Nanda AM, Thormann K, Frunzke J. Impact of spontaneous prophage induction on the fitness of bacterial populations and host-microbe interactions. *J Bacteriol* 2015;197:410–9. 10.1128/JB.02230-14 25404701 PMC4285972

[19] Little JW, Mount DW. The SOS regulatory system of *Escherichia coli*. *Cell* 1982;29:11–22. 10.1016/0092-8674(82)90085-X 7049397

[20] Melechen NE, Go G. Induction of lambdoid prophages by amino acid deprivation differential inducibility; role of *recA*. *Mol Gen Genet* 1980;180:147–55. 10.1007/BF00267364 6449654

[21] Tan D, Hansen MF, de Carvalho LN et al. High cell densities favor lysogeny: induction of an H20 prophage is repressed by quorum sensing and enhances biofilm formation in *vibrio anguillarum*. *ISME J* 2020;14:1731–42. 10.1038/s41396-020-0641-3 32269377 PMC7305317

[22] Glud RN, Berg P, Thamdrup B et al. Hadal trenches are dynamic hotspots for early diagenesis in the deep sea. *Commun Earth Environ* 2021;2:21. 10.1038/s43247-020-00087-2

[23] Oguri K, Masqué P, Zabel M et al. Sediment accumulation and carbon burial in four hadal trench systems. *J Geophys Res Biogeosci* 2022;127:e2022JG006814. 10.1029/2022JG006814

[24] Wenzhoefer F, Oguri K, Middelboe M et al. Benthic carbon mineralization in hadal trenches: assessment by *in situ* O2 microprofile measurements. Deep Sea res 1 Oceanogr res. *Pap* 2016;116:276–86. 10.1016/j.dsr.2016.08.013

[25] Jian H, Yi Y, Wang J et al. Diversity and distribution of viruses inhabiting the deepest ocean on earth. *ISME J* 2021;15:3094–110. 10.1038/s41396-021-00994-y 33972725 PMC8443753

[26] Sun X, Jiang H, Zhang S. Diversities and interactions of phages and bacteria in deep-sea sediments as revealed by metagenomics. *Front Microbiol* 2024;14:1337146. 10.3389/fmicb.2023.1337146 38260883 PMC10801174

[27] Corinaldesi C, Tangherlini M, Del-Anno A. From virus isolation to metagenome generation for investigating viral diversity in deep- sea sediments. Sci Rep 2017;7:8355. 10.1038/s41598-017-08783-4 28827715 PMC5566222

[28] Yoshida M, Yoshida-Takashima Y, Nunoura T et al. Identification and genomic analysis of temperate *pseudomonas* bacteriophage PstS-1 from the Japan trench at a depth of 7000 m. *Res Microbiol* 2015;166:668–76. 10.1016/j.resmic.2015.05.001 26025640

[29] Su Y, Zhang W, Liang Y et al. Identification and genomic analysis of temperate *Halomonas* bacteriophage vB_HmeY_H4907 from the surface sediment of the Mariana trench at a depth of 8,900 m. *Microbiol Spectr* 2023;11:0191223. 10.1128/spectrum.01912-23 PMC10580944 37728551

[30] Angel MV . Ocean trench conservation. *Environmentalist* 1982;2:1–17. 10.1007/BF02340472

[31] Schauberger C, Middelboe M, Larsen M et al. Spatial variability of prokaryotic and viral abundances in the Kermadec and Atacama trench regions. *Limnol Oceanogr* 2021;66:2095–109. 10.1002/lno.11711 34239169 PMC8248377

[32] Kropinski AM, Mazzocco A, Waddell TE et al. Enumeration of bacteriophages by double agar overlay plaque assay. Meth Mol Biol 2009;501:69–76. 10.1007/978-1-60327-164-6_7 19066811

[33] Middelboe M, Chan AM, Bertelsen SK. Isolation and life cycle characterization of lytic viruses infecting heterotrophic bacteria and cyanobacteria. *Man Aquat Vir Ecol* 2010;Chapt 13:118–33. 10.4319/mave.2010.978-0-9845591-0-7.118

[34] Mazzocco A, Waddell TE, Lingohr E et al. Enumeration of bacteriophages using the small drop plaque assay system. *Methods Mol Biol* 2009;501:81–5. 10.1007/978-1-60327-164-6_9 19066813

[35] Mauritzen JJ, Castillo D, Tan D et al. Beyond cholera: characterization of zot-encoding filamentous phages in the marine fish pathogen *vibrio anguillarum*. *Viruses* 2020;12:730. 10.3390/v12070730 32640584 PMC7412436

[36] Castillo D, Christiansen RH, Espejo R et al. Diversity and geographical distribution of *Flavobacterium psychrophilum* isolates and their phages: patterns of susceptibility to phage infection and phage host range. *Microb Ecol* 2014;67:748–57. 10.1007/s00248-014-0375-8 24557506

[37] Castillo D, Andersen N, Kalatzis PG et al. Large phenotypic and genetic diversity of prophages induced from the fish pathogen *Vibrio anguillarum*. *Viruses* 2019;11:983. 10.3390/v11110983 31653117 PMC6893619

[38] Shaffer M, Borton MA, McGivern BB et al. DRAM for distilling microbial metabolism to automate the curation of microbiome function. *Nucleic Acids Res* 2020;48:8883–900. 10.1093/nar/gkaa621 32766782 PMC7498326

[39] Prokka ST . Rapid prokaryotic genome annotation. *Bioinformatics* 2014;30:2068–9. 10.1093/bioinformatics/btu153 24642063

[40] Tamegai H, Li L, Masui N et al. A denitrifying bacterium from the deep sea at 11,000-m depth. *Extremophiles* 1997;1:207–11. 10.1007/s007920050035 9680302

[41] Wei Y, Mao H, Xu Y et al. *Pseudomonas abyssi* sp.Nov., isolated from the abyssopelagic water of the Mariana trench. *Int J Syst Evol Microbiol* 2018;68:2462–7. 10.1099/ijsem.0.002785 29927369

[42] Sun J, Wang W, Ying Y et al. *Pseudomonas profundi* sp. nov., isolated from deep-sea water. *Int J Syst Evol Microbiol* 2018;68:1776–80. 10.1099/ijsem.0.002748 29620498

[43] Yang Y, Gao Y, Liu Y et al. *Pseudomonas marianensis* sp. nov., a marine bacterium isolated from deep-sea sediments of the Mariana trench. *Arch Microbiol* 2022;204:638. 10.1007/s00203-022-03250-9 36131209

[44] Quigley MM, Colwell RR. Properties of bacteria isolated from deep-sea sediments. *J Bacteriol* 1968;95:211–20. 10.1128/jb.95.1.211-220.1968 5636819 PMC251994

[45] Gomila M, Peña A, Mulet M et al. Phylogenomics and systematics in *pseudomonas*. *Front Microbiol* 2015;6:214. 10.3389/fmicb.2015.00214 26074881 PMC4447124

[46] Katoh K, Misawa K, Kuma KI et al. MAFFT: a novel method for rapid multiple sequence alignment based on fast Fourier transform. *Nucleic Acids Res* 2002;30:3059–66. 10.1093/nar/gkf436 12136088 PMC135756

[47] Trouche B, Schauberger C, Bouderka F et al. Distribution and genomic variation of ammonia-oxidizing archaea in abyssal and hadal surface sediments. *ISME Comm* 2023;3:133. 10.1038/s43705-023-00341-6 PMC10746724 38135695

[48] Langmead B, Salzberg SL. Fast gapped-read alignment with bowtie 2. *Nat Methods* 2012;9:357–9. 10.1038/nmeth.1923 22388286 PMC3322381

[49] Li H, Handsaker B, Wysoker A et al. The sequence alignment/map format and SAMtools. *Bioinformatics* 2009;25:2078–9. 19505943 10.1093/bioinformatics/btp352 PMC2723002

[50] Kang DD, Li F, Kirton E et al. MetaBAT 2: an adaptive binning algorithm for robust and efficient genome reconstruction from metagenome assemblies. *PeerJ* 2019;7:e7359. 10.7717/peerj.7359 31388474 PMC6662567

[51] Stief P, Schauberger C, Becker KW et al. Hydrostatic pressure induces transformations in the organic matter and microbial community composition of marine snow particles. *Commun Earth Environ* 2023;4:377. 10.1038/s43247-023-01045-4

[52] Hood M . Comparison of four methods for measuring chitinase activity and the application of the 4-MUF assay in aquatic environments. *J Microbiol Methods* 1991;13:151–60. 10.1016/0167-7012(91)90015-I

[53] Alqarni B, Colley B, Klebensberger J et al. Expression stability of 13 housekeeping genes during carbon starvation of *Pseudomonas aeruginosa*. *J Microbiol Methods* 2016;127:182–7. 10.1016/j.mimet.2016.06.008 27297333

[54] Tan D, Dahl A, Middelboe M. Vibriophages differentially influence biofilm formation by *vibrio anguillarum* strains. *Appl Environ Microbiol* 2015;81:4489–97. 10.1128/AEM.00518-15 25911474 PMC4475874

[55] Girard L, Lood C, Höfte M et al. The ever-expanding pseudomonas genus: description of 43 new species and partition of the *pseudomonas putida* group. *Microorganisms* 2021;9:1766. 10.3390/microorganisms9081766 34442845 PMC8401041

[56] Liu Y, Zheng K, Liu B et al. Characterization and genomic analysis of Marinobacter phage vB_MalS-PS3, representing a new lambda-like temperate Siphoviral genus infecting algae-associated bacteria. *Front Microbiol* 2021;12:726074. 10.3389/fmicb.2021.726074 34512604 PMC8424206

[57] Thamdrup B, Schauberger C, Larsen M et al. Anammox bacteria drive fixed nitrogen loss in hadal trench sediments. *Proc Natl Acad Sci* 2021;118:e2104529118. 10.1073/pnas.2104529118 34764222 PMC8609620

[58] Quigley MM, Colwell RR. Proposal of a new species of *pseudomonas bathycetes*. *Int J Syst Bacteriol* 1968;18:241–52. 10.1099/00207713-18-3-241

[59] Schauberger C, Glud RN, Hausmann B et al. Microbial community structure in hadal sediments: high similarity along trench axes and strong changes along redox gradients. *ISME J* 2021;15:3455–67. 10.1038/s41396-021-01021-w 34103697 PMC8629969

[60] Kampers LFC, Koehorst JJ, van Heck RJA et al. A metabolic and physiological design study of *pseudomonas putida* KT2440 capable of anaerobic respiration. *BMC Microbiol* 2021;21:9. 10.1186/s12866-020-02058-1 33407113 PMC7789669

[61] Tian Y, Cai L, Xu Y et al. Stability and infectivity of allochthonous viruses in deep sea: a long-term high pressure simulation experiment. *Deep Sea Res 1 Oceanogr Res Pap* 2020;161:103302. 10.1016/j.dsr.2020.103302

[62] Souza CP, Almeida BC, Colwell RR et al. The importance of chitin in the marine environment. *Mar Biotechnol* 2011;13:823–30. 10.1007/s10126-011-9388-1 21607543

[63] Castillo D, Rørbo N, Jørgensen J et al. Phage defense mechanisms and their genomic and phenotypic implications in the fish pathogen *vibrio anguillarum*. *FEMS Microbiol Ecol* 2019;95:fiz004. 10.1093/femsec/fiz004 30624625

[64] Gödeke J, Paul K, Lassak J et al. Phage-induced lysis enhances biofilm formation in *Shewanella oneidensis* MR-1. *ISME J* 2011;5:613–26. 10.1038/ismej.2010.153 20962878 PMC3105746

[65] Hansen MF, Svenningsen SL, Røder HL et al. Big impact of the tiny: bacteriophage–bacteria interactions in biofilms. *Trends Microbiol* 2019;27:739–52. 10.1016/j.tim.2019.04.006 31128928

[66] Tan D, Lo SS, Middelboe M. Quorum sensing determines the choice of antiphage defense strategy in *vibrio anguillarum*. *MBio* 2015;6:e00627. 10.1128/mBio.00627-15 26081633 PMC4471561

[67] Erez Z, Steinberger-Levy I, Shamir M et al. Communication between viruses guides lysis–lysogeny decisions. *Nature* 2017;541:488–93. 10.1038/nature21049 28099413 PMC5378303

[68] Mara P, Vik D, Pachiadaki MG et al. Viral elements and their potential influence on microbial processes along the permanently stratified Cariaco Basin redoxcline. *ISME J* 2020;14:3079–92. 10.1038/s41396-020-00739-3 32801311 PMC7785012

[69] Breitbart M, Bonnain C, Malki K et al. Phage puppet masters of the marine microbial realm. *Nat Microbiol* 2018;3:754–66. 10.1038/s41564-018-0166-y 29867096

[70] Tuttle MJ, Buchan A. Lysogeny in the oceans: lessons from cultivated model systems and a reanalysis of its prevalence. *Environ Microbiol* 2020;22:4919–33. 10.1111/1462-2920.15233 32935433

[71] Clokie MRJ, Shan J, Bailey S et al. Transcription of a ‘photosynthetic’ T4-type phage during infection of a marine cyanobacterium. *Environ Microbiol* 2006;8:827–35. 10.1111/j.1462-2920.2005.00969.x 16623740

