# Supplementary material for: Prophage-encoded chitinase gene supports growth of its bacterial host isolated from deep-sea sediments
Source: ISME J. 2025 Jan 20;19(1):wraf004. doi: 10.1093/ismejo/wraf004 (PMC11788074; doi:10.1093/ismejo/wraf004)
Supplement: Middelboe_et_al_suppl_mat_Submission_ISME_rev_09012025_wraf004 [file middelboe_et_al_suppl_mat_submission_isme_rev_09012025_wraf004.pdf]

Supplementary material

Prophage-encoded chitinase gene supports growth of its bacterial host isolated from deep-sea sediments

Mathias Middelboe, Sachia J. Traving, Daniel Castillo, Panos G. Kalatzis, Ronnie N. Glud

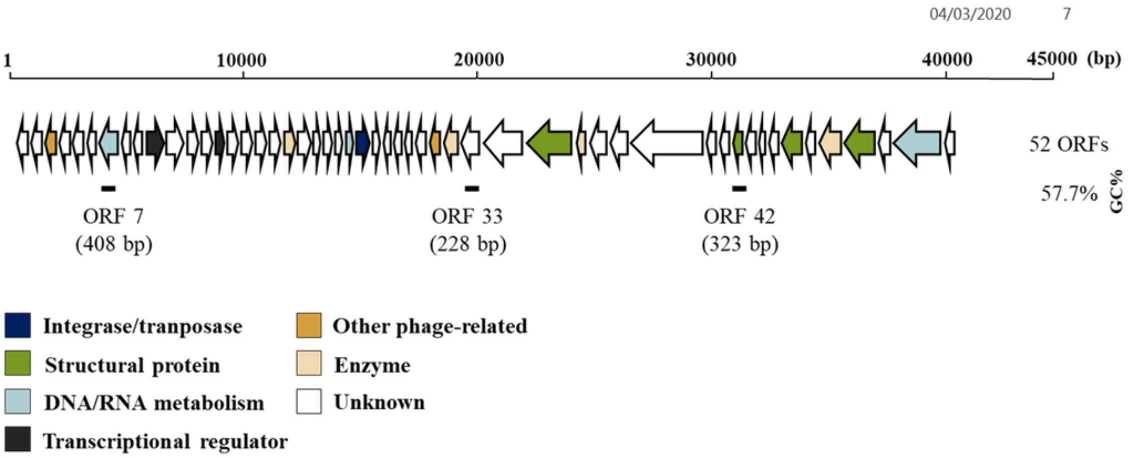

Figure S1: Genome organization of phage Pseudomonas phage_KT1. ORFs 6, 33 and 42 were the target genes for identification of phage_KT1 in *Pseudomonas* isolates.

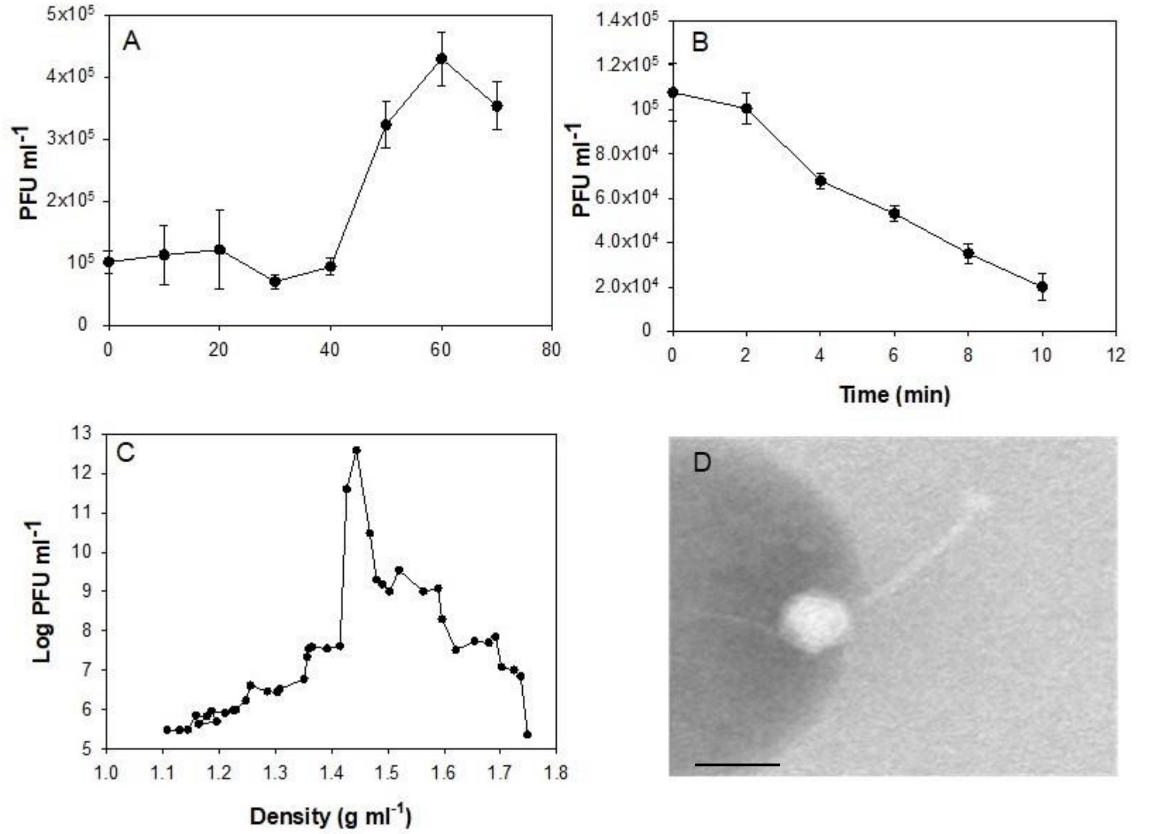

Figure S2: A) One-step growth experiment with phage Pseudomonas phage_KT1 and host KT_2-4 WT B) Decrease in free phage Pseudomonas phage_KT1 concentration over time during the adsorption experiment with the host *Pseudomonas* sp KT_2-4 (WT). C) Concentration of the infective phage Pseudomonas phage_KT1 as a function of solute density during density-gradient centrifugation. D) Transmission electron micrograph of Pseudomonas phage_KT1 (Scale bar: 50 nm).

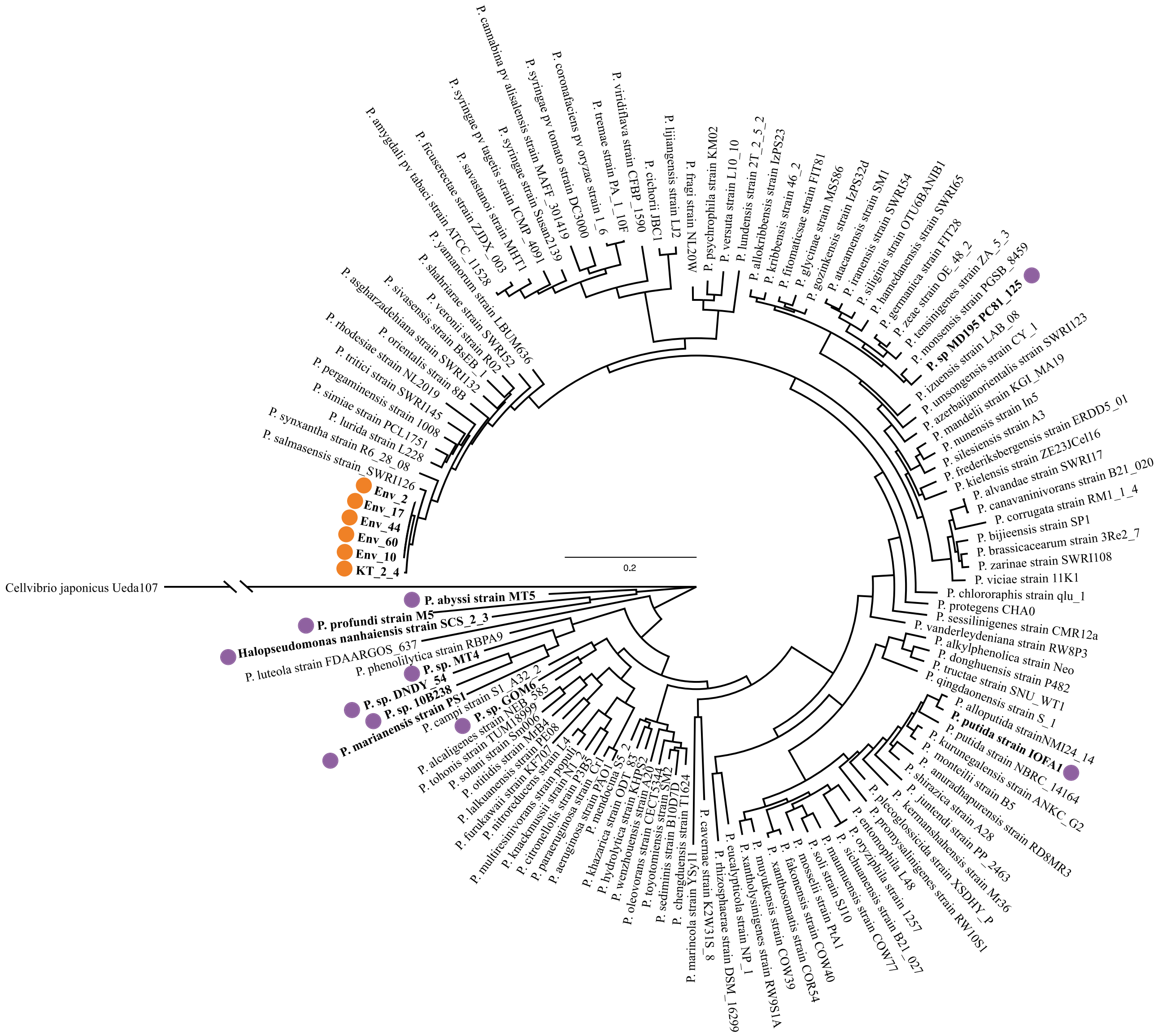

Figure S3: Maximum-likelihood tree of multi-sequence alignment of the four genes 16S rRNA gene, *rpoD*, *rpoB* and gyrB for the six *Pseudomonas* sp. isolates from the present study (orange), 113 *Pseudomonas* reference genomes and 10 (purple) *Pseudomonas* isolates from deep sea or hadal environments. *Cellvibrio japonicum* Ueda107m is used as the outgroup, and the branch length has been truncated for visual purposes.

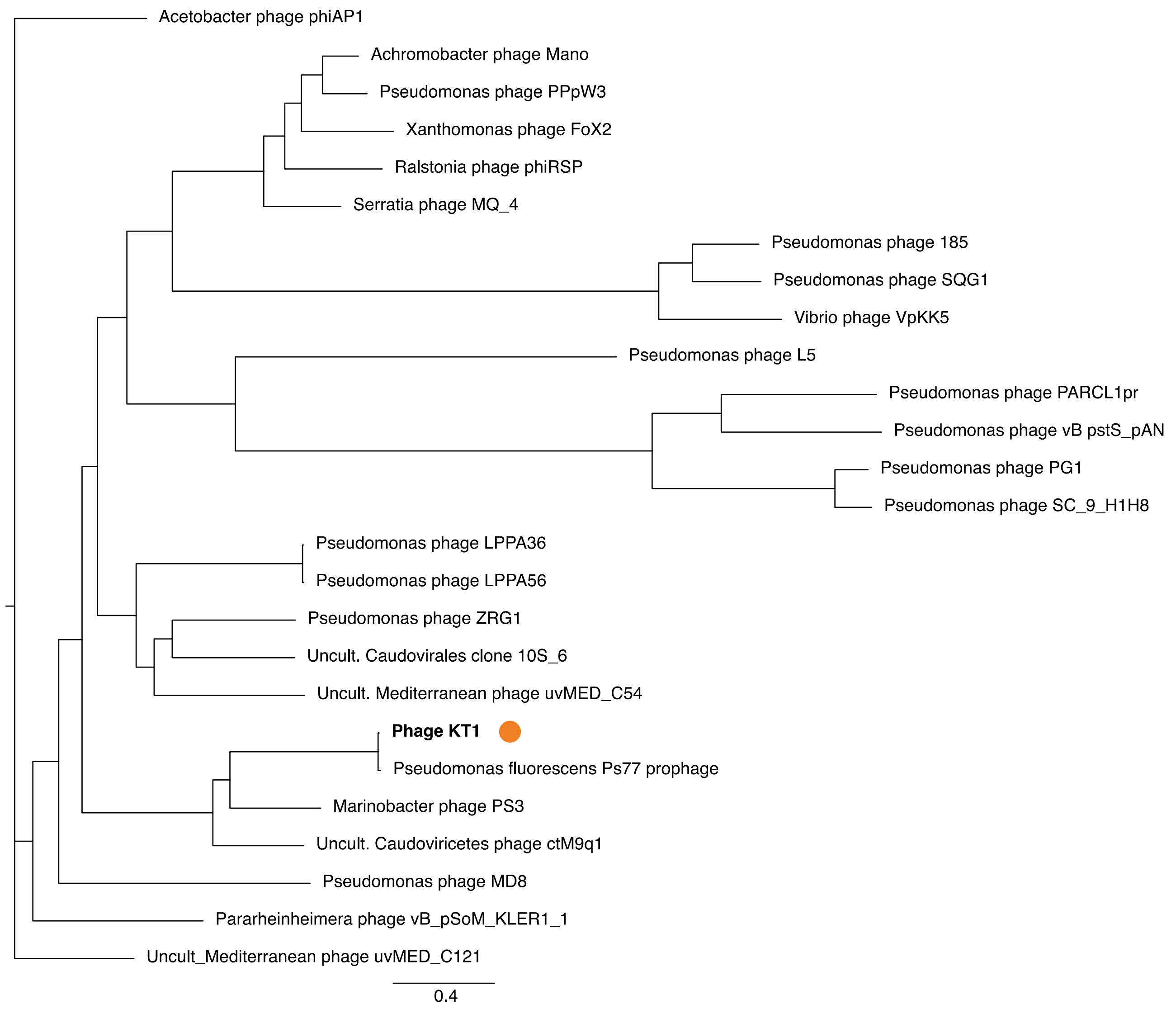

Figure S4: Maximum-likelihood tree of the phage isolated in this study (orange) and closest found relatives based on the alignment of the terminase large subunit gene.

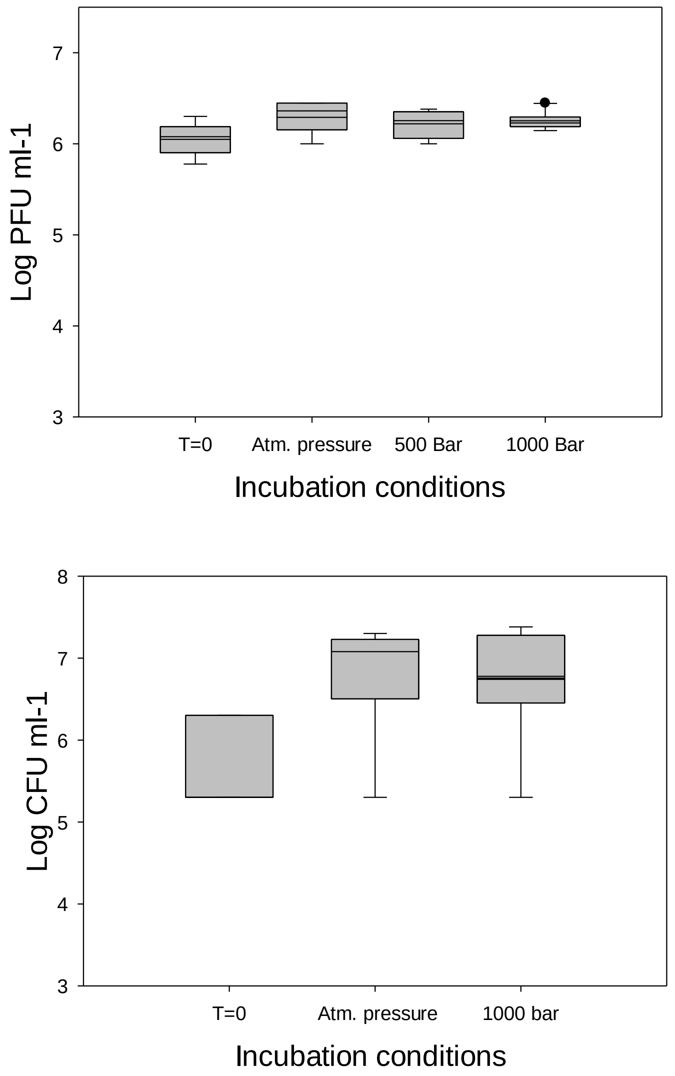

Figure S5: Effects of hydrostatic pressure on phage infectivity and host cultivability. Abundance of A) infective Pseudomonas phage_KT1 phages (PFU ml^{-1}) and B) culturable *Pseudomonas* sp. KT_2-4 WT (CFU ml^{-1}) before and after 72 h exposure to elevated hydrostatic pressure in separate experiments.

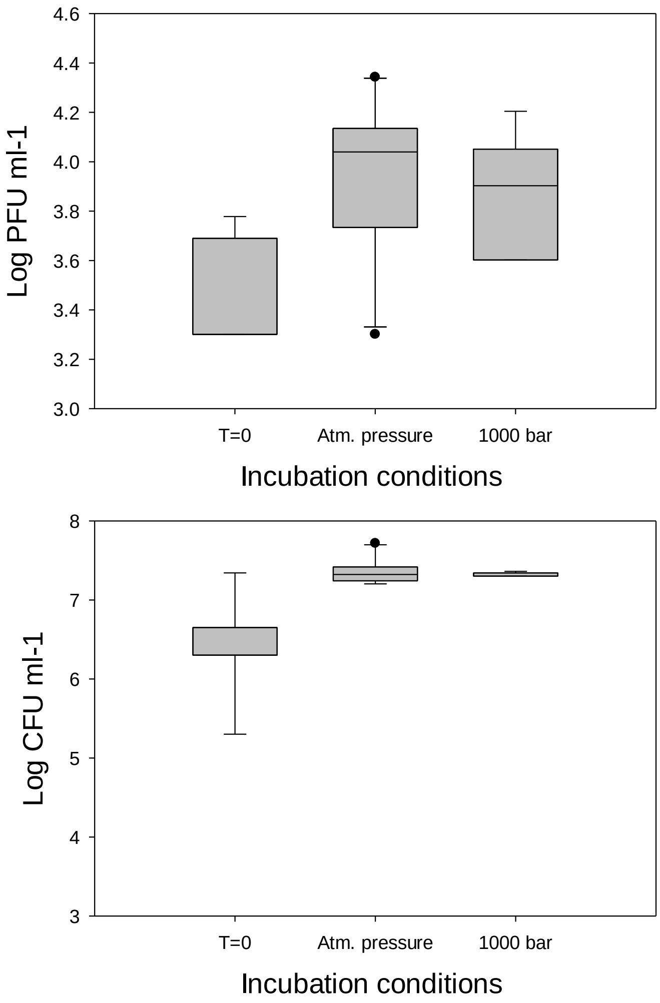

Figure S6: Effect of hydrostatic pressure on phage-host interactions. Abundance of A) infective Pseudomonas phage_KT1 phages (PFU ml^{-1}) and B) culturable Pseudomonas sp. KT_2-4 WT (CFU ml^{-1}) before and after 72 h exposure to elevated hydrostatic pressure when inoculated together.

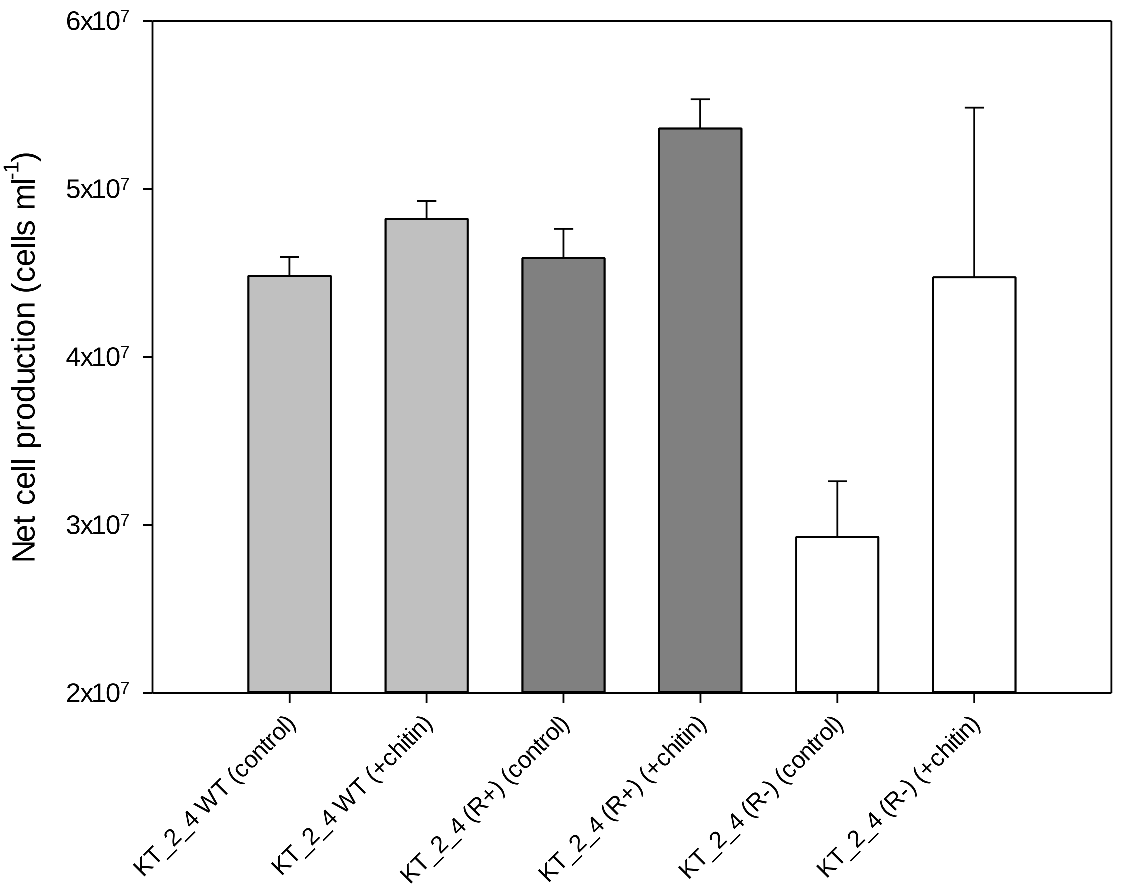

Figure S7: Net cell production of WT, R+, and R− strains during growth in chitin-enriched medium and control medium without chitin in batch culture experiments.

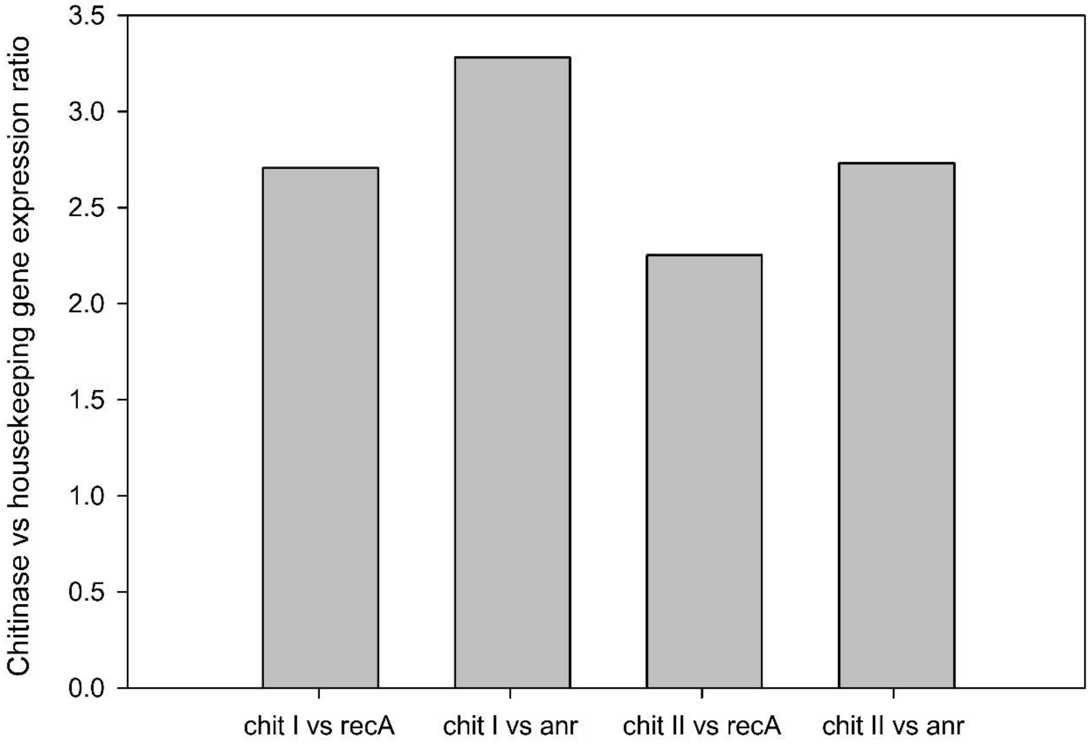

Figure S8: qRT PCR data from expression analysis of the prophage encoded chitinase gene in the presence of chitin. The bars show the fold increase in the expression level of the prophage encoded chitinase when growing in the presence of chitin compared to control condition without chitin. Each bar represents the fold increase using different combinations of the two primers for the target gene (*chitI* and *chitII*) and the two housekeeping genes (*recA* and *anr*).

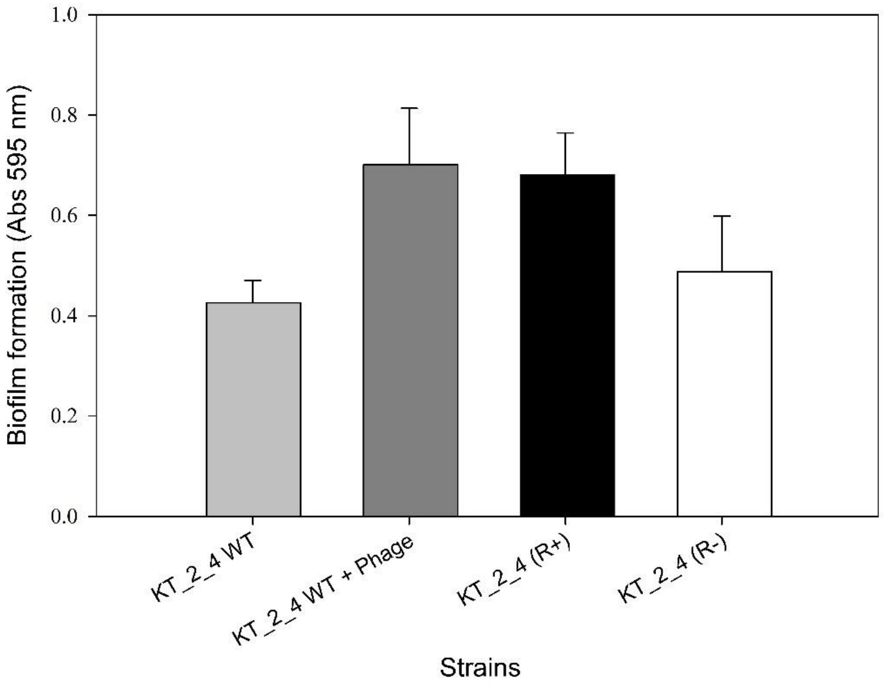

Figure S9: Biofilm formation by the three strains WT, R+, and R-, as well as by the WT strain in the presence of phage Pseudomonas phage_KT1.

Table S1: List of environmental isolates from Kermadec trench sites 4 and 5 and reference site 7 with indications of sediment depth (cm). Black squares indicate the presence of the individual prophage genes DNA polymerase (ORF 7), hypothetical protein (ORF 33) and structural protein (ORF 42) by PCR. The results of the sensitivity test against Pseudomonas phage_KT1 are shown in column 6.

Isolate	Site/Depth (cm)	PCR target			Phage susceptibility
		DNA polym.	Hypoth. protein	Struct. protein	
1	Site 4/0-1	■	■	■	Resistant
2	Site 4/0-1	■	■	■	Resistant
3	Site 4/0-1	■	■	■	Resistant
4	Site 4/0-1	■	■	■	Resistant
5	Site 4/1-2	■	■	■	Resistant
6	Site 4/1-2	■	■	■	Resistant
7	Site 4/1-2	■	■	■	Resistant
8	Site 4/2-4				Sensitive
9	Site 4/2-4				Sensitive
10	Site 4/2-4	■	■	■	Resistant
11	Site 4/2-4	■	■	■	Sensitive
12	Site 4/4-6	■	■	■	Resistant
13	Site 4/4-6	■	■	■	Resistant
14	Site 4/4-6	■	■	■	Resistant
15	Site 4/6-8	■	■	■	Resistant
16	Site 4/6-8	■	■	■	Resistant
17	Site 4/6-8	■	■	■	Resistant
18	Site 4/8-10	■	■	■	Resistant
19	Site 4/8-10	■	■	■	Resistant
20	Site 4/8-10	■	■	■	Resistant
21	Site 4/10-15				Resistant
22	Site 4/15-20	■	■	■	Resistant
23	Site 4/15-20	■	■	■	Resistant
24	Site 4/15-20	■	■	■	Resistant
25	Site 4/20-25	■	■	■	Resistant
26	Site 4/20-25	■	■	■	Resistant
27	Site 4/20-25	■	■	■	Resistant
28	Site 4/20-25	■	■	■	Resistant
29	Site 4/25-30	■	■	■	Resistant
30	Site 4/25-30	■	■	■	Resistant
31	Site 4/25-30	■	■	■	Resistant
32	Site 4/25-30	■	■	■	Resistant
33	Site 5/1-2	■	■	■	Resistant
34	Site 5/1-2	■	■	■	Resistant
35	Site 5/1-2	■	■	■	Resistant
36	Site 5/1-2	■	■	■	Resistant
37	Site 5/2-4	■	■	■	Resistant
38	Site 5/2-4	■	■	■	Resistant
39	Site 5/4-6	■	■	■	Resistant
40	Site 5/4-6	■	■	■	Resistant
41	Site 5/4-6	■	■	■	Resistant
42	Site 5/6-8	■	■	■	Resistant
43	Site 5/6-8	■	■	■	Resistant
44	Site 5/6-8	■	■	■	Resistant

45	Site 5/8-10	[redacted]	[redacted]	[redacted]	Resistant
46	Site 5/8-10	[redacted]	[redacted]	[redacted]	Resistant
47	Site 5/8-10	[redacted]	[redacted]	[redacted]	Resistant
48	Site 5/10-15	[redacted]	[redacted]	[redacted]	Resistant
49	Site 5/10-15	[redacted]	[redacted]	[redacted]	Resistant
50	Site 5/10-15	[redacted]	[redacted]	[redacted]	Resistant
51	Site 5/15-20	[redacted]	[redacted]	[redacted]	Resistant
52	Site 5/15-20	[redacted]	[redacted]	[redacted]	Resistant
53	Site 5/15-20	[redacted]	[redacted]	[redacted]	Resistant
54	Site 5/20-25	[redacted]	[redacted]	[redacted]	Resistant
55	Site 5/20-25	[redacted]	[redacted]	[redacted]	Resistant
56	Site 5/20-25	[redacted]	[redacted]	[redacted]	Resistant
57	Site 5/25-30	[redacted]	[redacted]	[redacted]	Resistant
58	Site 5/25-30	[redacted]	[redacted]	[redacted]	Resistant
59	Site 5/25-30	[redacted]	[redacted]	[redacted]	Resistant
60	Site 5/25-30	[redacted]	[redacted]	[redacted]	Resistant
61	Site 5/30-35	[redacted]	[redacted]	[redacted]	Resistant
62	Site 5/30-35	[redacted]	[redacted]	[redacted]	Resistant
63	Site 5/30-35	[redacted]	[redacted]	[redacted]	Resistant
64	Site 5/30-35	[redacted]	[redacted]	[redacted]	Resistant
65	Site 7/0-1				Resistant
66	Site 7/0-1	[redacted]	[redacted]	[redacted]	Resistant
67	Site 7/0-1				Resistant
68	Site 7/0-1	[redacted]	[redacted]	[redacted]	Resistant
69	Site 7/1-2	[redacted]	[redacted]	[redacted]	Resistant
70	Site 7/1-2	[redacted]	[redacted]	[redacted]	Resistant
71	Site 7/1-2	[redacted]	[redacted]	[redacted]	Resistant
72	Site 7/1-2	[redacted]	[redacted]	[redacted]	Resistant
73	Site 7/4-6	[redacted]	[redacted]	[redacted]	Resistant
74	Site 7/4-6	[redacted]	[redacted]	[redacted]	Resistant
75	Site 7/4-6	[redacted]	[redacted]	[redacted]	Resistant
76	Site 7/4-6	[redacted]	[redacted]	[redacted]	Resistant
77	Site 7/6-8	[redacted]	[redacted]	[redacted]	Resistant
78	Site 7/6-8	[redacted]	[redacted]	[redacted]	Resistant
79	Site 7/6-8	[redacted]	[redacted]	[redacted]	Resistant
80	Site 7/6-8	[redacted]	[redacted]	[redacted]	Resistant
81	Site 7/8-10	[redacted]	[redacted]	[redacted]	Resistant
82	Site 7/8-10	[redacted]	[redacted]	[redacted]	Resistant
83	Site 7/8-10	[redacted]	[redacted]	[redacted]	Resistant
84	Site 7/8-10	[redacted]	[redacted]	[redacted]	Resistant
85	Site 7/10-15	[redacted]	[redacted]	[redacted]	Resistant
86	Site 7/10-15	[redacted]	[redacted]	[redacted]	Resistant
87	Site 7/10-15	[redacted]	[redacted]	[redacted]	Resistant
88	Site 7/10-15	[redacted]	[redacted]	[redacted]	Resistant
89	Site 7/10-15	[redacted]	[redacted]	[redacted]	Resistant
90	Site 7/10-15	[redacted]	[redacted]	[redacted]	Resistant
91	Site 7/15-20	[redacted]	[redacted]	[redacted]	Resistant
92	Site 7/15-20	[redacted]	[redacted]	[redacted]	Resistant
93	Site 7/15-20	[redacted]	[redacted]	[redacted]	Resistant
94	Site 7/15-20	[redacted]	[redacted]	[redacted]	Resistant
95	Site 7/15-20	[redacted]	[redacted]	[redacted]	Resistant
96	Site 7/15-20	[redacted]	[redacted]	[redacted]	Sensitive

Table S2: Calculating of the per sample coverage of the Pseudomonas isolate in metagenomic samples.

There were two sampling stations (Hadal and abyssal reference site), with one metagenomic sample per depth. Created index from the *Pseudomonas* isolate genome using Bowtie2 (2.2.5)

```
bowtie2-build ~/Pseudomonas/DS-C6_genome.fasta pseudomonas_bw_index
```

Mapping QC'ed reads to the index using Bowtie2.

```
for i in ~/01_QC/A_HADES_Kermadec_*.fastq.gz; do
  sample_name=$(basename "$i" .fastq.gz)
  base_name=${sample_name%_R*}
  bowtie2 -x ~/Pseudomonas/pseudomonas_bw_index \
  -1 "~/01_QC/${base_name}_R1.fastq.gz" \
  -2 "~/01_QC/${base_name}_R2.fastq.gz" \
  -S "~/Pseudomonas/coverage/alignment_${sample_name}.sam" --threads 32
done
```

Converting to .bam file using Samtools (2.17)

```
for i in ~/Pseudomonas/coverage/alignment_*.sam; do
  samtools sort "$i" -o "${i%.sam}.sorted.bam"
```

Using the jgi_summarize function from MetaBAT2 (2.2.15) to calculate the coverage table.

```
jgi_summarize_bam_contig_depths --outputDepth coverage.txt
~/Pseudomonas/coverage/alignment_*.sorted.bam
```

The library size file for normalizing each sample was created as follows:

```
for i in ~/Pseudomonas/coverage/*.bam; do
  filename=$(basename "$i")
  libsize=$(($(samtools flagstat "$i" | head -n 1 | cut -f 1 -d ' ')/2))
  printf "%s\t%d\n" "$filename" "$libsize" >> libsize.txt
done
```

Then the custom script created by D. Senanayake was applied. The script can be found here: https://genomicsaotearoa.github.io/metagenomics_summer_school/resources/3_APPENDIX_ex11_Normalise_coverage_example/#2-normalise-and-scale-contig-coverage

in R:

```
./normalize_jgi_cov.r coverage.txt libsize.txt
```

Table S3: List of isolates from the infection experiment obtained after 12 h of phage exposure with indication of sensitivity or resistance to the Pseudomonas phage_KT1 phage, the presence of the phage as a prophage, and efficiency of plating relative to the WT strain. *indicates a highly reduced sensitivity to the phage relative to the wild type strain.

	Phenotype	Lysogenic	EOP
WT	Sensitive	No	1
C1	Resistant	No	0
C2	Resistant	Yes	0
C3	Sentitive	No	1
C4	Resistant	Yes	0
C5	Sentitive	No	1
C6	Resistant	Yes	0
C7	Resistant	Yes	0
C8	Resistant	Yes	0
C9	Resistant	nd	0
C10	Resistant	nd	0
C11	Sentitive	No	1
C12	Sentitive	No	1
C13	Resistant	nd	0
C14	Resistant	nd	0
C15	Resistant	nd	0
C16	Sentitive	No	1
C17	Resistant	nd	0
C18	Resistant	nd	0
C19	Sentitive	No	1
C20	Resistant	nd	0
C21	Resistant	nd	0
C22	Sentitive	No	1
C23	Resistant	nd	0
C24	Resistant	nd	0
C25	Resistant	nd	0
C26	Resistant	nd	0
C27	Resistant	nd	0
C28	Sensitive	No	1
C29	Sensitive	No	1
C30	Resistant	Yes	0
C31	Sensitive	No	1
C32	Resistant	Yes	0
C33	Resistant	Yes	0
C34	Resistant	Yes	0
C35	Resistant	Yes	0
C36	Resistant	Yes	0
C37	Sensitive	No	1
C38	Sensitive	No	1
C39	Resistant	Yes	0

C40	Resistant	Yes	0
C41	Resistant	Yes	0
C42	Resistant	Yes	0
C43	Resistant	Yes	0
C44	Resistant	Yes	0
C45	Resistant	Yes	0
C46	Sensitive	No	1
C47	Sensitive	No	1
C48	Sensitive	No	1
C49	Sensitive	No	1
C50	Resistant	Yes	0
C51	Resistant	Yes	0
C52	Sensitive	No	1
C53	Resistant	Yes	0
C54	Resistant	Yes	0
C55	Sensitive	No	1
C56	Resistant	Yes	0
C57	Resistant	Yes	0
C58	Resistant	Yes	0
C59	Sensitive	No	1
C60	Sensitive	No	1
C61	Sensitive	No	1
C62	Resistant	Yes	0
C63	Resistant	Yes	0
C64	Sensitive*	nd	1.00E-07
C65	Sensitive	No	1
C66	Resistant	Yes	0
C67	Sensitive	No	1
C68	Resistant	Yes	0
C69	Resistant	Yes	0
C70	Resistant	Yes	0
C71	Sensitive	No	1
C72	Resistant	Yes	0
C73	Sensitive*	No	1.00E-07
C74	Sensitive*	No	1.00E-07
C75	Resistant	Yes	0
C76	Resistant	Yes	0
C77	Sensitive	No	1
C78	Sensitive	No	1
C79	Sensitive	No	1
C80	Resistant	Yes	0
C81	Sensitive	No	1
C82	Sensitive	No	1
C83	Resistant	Yes	0
C84	Sensitive*	ND	1.00E-07
C85	Resistant	Yes	0
C86	Resistant	Yes	0
C87	Sensitive	No	1
C88	Sensitive	No	0.92
C89	Resistant	Yes	0

C90	Resistant	Yes	0
C91	Sensitive	No	1
C92	Resistant	Yes	0
C93	Resistant	Yes	0
C94	Sensitive	No	1
C95	Resistant	Yes	0
C96	Sensitive	No	0.97
C97	Sensitive*	nd	1.00E-07
C98	Resistant	Yes	0
C99	Resistant	Yes	0
C100	Resistant	Yes	0

Table S4: The primer sequences used for detection of Pseudomonas phage_KT1 phage as a prophage in the collection of *Pseudomonas* KT_2_4 host isolates.

**Target gene**	**Tm (°C)**	**Primer sequences (5´→3´)**	**Product size (bp)**
DNA polymerase (ORF 7)	60	Forward: CACGCTTGATAGGTTTCGCG<br>Reverse: AGCACAGCGATATCGTCCTG	748
Hypothetical protein (ORF 33)	60	Forward: CATAGACACCGTCGACCTCG<br>Reverse: ACCGTTATCAGTGGCTGCAA	505
Putative structural protein (ORF2)	60.1	Forward: AATATCCTGGGGCGTGGTTG<br>Reverse: GTGGGGTTGTTTTACGCCACC	293

Table S5: Annotation of Pseudomonas phage_KT1

ORF	Nucleotide position	Predicted function	BLASTP most significant match (accession; E- value)	% amino acid identity	Conserved domain database hit E-value<0.001 (accession)
1	134-865	Unknown	Hypothetical protein [*Pseudomonas* sp. Eur1 9.41] (WP_032892958.1; 6e-174)	98.7	None
2	926-1207	Unknown	None	None	None
3	1307-1654	Holin	Phage holin family protein [*Pseudomonas* sp. NBRC 111138] (WP_054917674.1; 2e-60)	98	Putative 3TM holing (pfam05449)
4	1590-1982	Unknown	Hypothetical protein [*Pseudomonas*] (WP_056783444.1; 2e-77)	100	Putative phage holin (pfam16931)
5	2215-2703	Unknown	Hypothetical protein [*Pseudomonas veronii*] (WP_126585627.1; 2e-112)	98	None
6	2722-3048	Unknown	Hypothetical protein [*Pseudomonas* sp. 286] (WP_122763743.1; 6e-15)	45.5	Cysteinyl-tRNA synthetase (COG0215)
7	3309-6122	DNA polymerase	Bifunctional DNA primase/polymerase [*Pseudomonas* sp. NFR09] (WP_093121098.1; 0)	70	Bifunctional DNA primase/polymeras e (pfam09250)
8	6225-6713	Unknown	Hypothetical protein [*Pseudomonas fluorescens*] (WP_106118331.1; 3e-115)	100	None
9	6738-7091	Unknown	Hypothetical protein PMI35_01240 [*Pseudomonas* sp. GM78] (EJN31751.1; 3e-08)	72	None
10	7181-8062	Transcriptional regulator	XRE family transcriptional regulator [*Pseudomonas fluorescens*] (WP_106118333.1; 0)	99	Phage repressor protein C (COG2932)
11	8380-8979	Unknown	Hypothetical protein [*Pseudomonas synxantha*]	98	deoxynucleoside monophosphate kinase (PHA02575)

			(WP_057024003.1; 5e-139)		
12	8976-9266	Unknown	Hypothetical protein [*Pseudomonas synxantha*] (WP_057024004.1; 4e-60)	100	Phage antirepressor protein KilAC domain (pfam03374)
13	9263-9637	Unknown	Hypothetical protein [*Pseudomonas synxantha*] (WP_057024005.1; 7e-80)	96	None
14	9646-9876	Transcriptional regulator	Transcriptional regulator [*Pseudomonas*] (WP_032892969.1; 7e-48)	98	Pyocin activator protein PrtN (pfam11112)
15	9892-10290	Unknown	Hypothetical protein [*Pseudomonas fluorescens* group] (WP_103451420.1; 7e-80)	100	None
16	10193-10609	Unknown	None	None	None
17	10335-11144	Unknown	DUF2303 family protein [*Pseudomonas fluorescens* group] (WP_103451421.1; 0)	100	Uncharacterized conserved protein (DUF2303) (pfam10065)
18	11030-11677	Unknown	Hypothetical protein [*Pseudomonas fluorescens* group] (WP_103451422.1; 5e-105)	100	None
19	11674-12219	Phosphohydrolase	Phosphohydrolase [*Pseudomonas fluorescens* group] (WP_103451423.1; 5e-132)	100	5'-deoxynucleotidase YfbR and related HD superfamily hydrolases (COG1896)
20	11964-13022	Unknown	DUF2786 domain-containing protein [*Pseudomonas fluorescens* group] (WP_103451424.1; 0)	100	Protein of unknown function (DUF2786) (pfam10979)
21	13019-13300	Unknown	Hypothetical protein [*Pseudomonas fluorescens* group] (WP_103451425.1; 1e-161)	100	None
22	13204-13641	Unknown	Hypothetical protein [*Pseudomonas fluorescens* group] (WP_103451426.1; 5e-75)	100	None
23	13499-13894	Unknown	Hypothetical protein [*Pseudomonas fluorescens* group] (WP_103451427.1; 6e-59)	100	None

24	14022-14294	DNA binding	DNA-binding protein [*Pseudomonas*] (WP_032892978.1; 3e-46)	100	Putative excisionase (DUF1233) (pfam06806)
25	14273-15508	Integrase	Site-specific integrase [*Pseudomonas fluorescens* group] (WP_103451428.1; 0)	100	C-terminal catalytic domain of integrases from bacterial phages and conjugate transposons (cd01189)
26	15733-15996	Unknown	None	None	None
27	15899-16168	Unknown	Hypothetical protein [*Pseudomonas fluorescens* group] (WP_103451387.1; 4e-57)	100	None
28	16165-16434	Unknown	Hypothetical protein [*Pseudomonas libanensis*] (WP_125969675.1; 1e-58)	100	None
29	16413-16697	Unknown	Hypothetical protein [*Pseudomonas fluorescens* group] (WP_103451388.1; 5e-64)	100	None
30	16852-17379	Unknown	Hypothetical protein [*Pseudomonas fluorescens* group] (WP_103451389.1; 2e-127)	99	None
31	17793-18299	Lysosyme	Lysis protein [*Pseudomonas fluorescens* group] (WP_103451390.1; 2e-115)	99	None
32	18302-18847	Hydrolase	Glycoside hydrolase family 19 protein [*Pseudomonas fluorescens* group] (WP_103451391.1; 1e-125)	100	Predicted chitinase (COG3179)
33	18910-20604	Unknown	Hypothetical protein [*Pseudomonas fluorescens* group] (WP_103451392.1; 0)	100	None
34	20728-23097	Unknown	Hypothetical protein [*Pseudomonas fluorescens*] (WP_103451393.1; 0)	100	None
35	23110-25722	Unknown	Hypothetical protein [*Pseudomonas fluorescens* group] (WP_103451394.1; 0)	99	Putative phage tail protein (pfam13550)
36	25631-26092	Unknown	CHAP domain-containing protein [*Pseudomonas fluorescens* group]	100	Putative phage cell wall peptidase, NlpC/P60 family (TIGR02219)

			(WP_103451395.1; 2e-107)		
37	26089-27039	Unknown	DUF2163 domain-containing protein [*Pseudomonas* sp. Eur1 9.41] (WP_084570939.1; 0)	95	Uncharacterized conserved protein (DUF2163) (pfam09931)
38	26949-27596	Unknown	Hypothetical protein [*Pseudomonas fluorescens* group] (WP_103451397.1; 1e-157)	100	Conserved hypothetical protein 2217 (DUF2460) (pfam09343)
39	27606-30377	Unknown	Hypothetical protein [*Pseudomonas fluorescens* group] (WP_103451398.1; 0)	100	Protein of unknown function (DUF3584) (pfam12128)
40	30445-30984	Unknown	Hypothetical protein [*Pseudomonas fluorescens* group] (WP_103451399.1; 2e-74)	100	None
41	31084-31446	Unknown	Hypothetical protein TU73_13200 [*Pseudomonas libanensis*] (KRP45358.1; 5e-75)	98	None
42	31428-32183	Putative structural protein	Hypothetical protein [*Pseudomonas fluorescens* group] (WP_103451401.1; 1e-180)	100	Phage major tail protein, phi13 family (TIGR01603)
43	32194-32646	Unknown	Hypothetical protein [*Pseudomonas fluorescens* group] (WP_103451402.1; 4e-105)	100	None
44	32650-32937	Unknown	Hypothetical protein [*Pseudomonas fluorescens* group] (WP_057012588.1; 2e-61)	100	None
45	33120-33497	Unknown	Hypothetical protein [*Pseudomonas fluorescens* group] (WP_057012590.1; 4e-52)	100	None
46	33403-34350	Putative structural protein	Hypothetical protein [*Pseudomonas fluorescens* group] (WP_103451403.1; 0)	100	Mu-like prophage major head subunit gpT (pfam10124)
47	34406-34840	Unknown	Cytoplasmic protein [*Pseudomonas fluorescens* group] (WP_103451404.1; 1e-90)	100	None
48	34864-36213	Peptidase	S49 family peptidase [*Pseudomonas fluorescens* group] (WP_103451405.1; 0)	100	Signal peptide peptidase A (SppA) (cd07022)

49	36216-37286	Structural	Phage portal protein [*Pseudomonas fluorescens* group] (WP_103451406.1; 0)	100	Phage portal protein, lambda family (pfam05136)
50	37823-38044	Unknown	Hypothetical protein [*Pseudomonas*] (WP_032892955.1; 6e-45)	100	None
51	38047-4170	Terminase	Phage terminase large subunit (GpA) [*Pseudomonas fluorescens*] (KWV70064.1; 0)	98	Phage terminase large subunit (GpA) (pfam05876)
52	40076-40570	Unknown	Hypothetical protein [*Pseudomonas fluorescens* group] (WP_103451407.1; 1e-114)	100	None

Table S6: The strains of *Pseudomonas* sp selected for experimental work

**Isolate**	**Description**
**Pseudomonas phage_KT1**	Temperate phage infecting *Pseudomonas* sp. KT_2-4 WT
***Pseudomonas* sp. KT_2_4 WT**	The non-lysogenic, phage sensitive wild type (WT) bacterial isolate obtained from Site 4 at 2-4 cm depth in the Kermadec Trench.
***Pseudomonas* sp. KT_2_4 (R-)**	Phage resistant, non-lysogenized isolate of the WT strain obtained in culture upon exposure to phage Pseudomonas phage_KT1
***Pseudomonas* sp. KT_2_4 (R+)**	Lysogenized isolate of WT strain obtained after exposure to phage Pseudomonas phage_KT1.
